# Morphological basis of the lung adenocarcinoma subtypes

**DOI:** 10.1016/j.isci.2024.109742

**Published:** 2024-04-12

**Authors:** Linjun Zha, Toru Matsu-ura, James P. Sluka, Tomohiro Murakawa, Koji Tsuta

**Affiliations:** 1 Department of Pathology, Kansai Medical University, Hirakata, Osaka 573-0033, Japan; 2 Biocomplexity Institute, Indiana University, Bloomington, IN 47405-7105, USA; 3 Department of Thoracic Surgery, Kansai Medical University, Hirakata, Osaka 573-0033, Japan

**Keywords:** Biological sciences, Cell biology, Cell, Cancer

## Abstract

Lung adenocarcinoma (LUAD), which accounts for a large proportion of lung cancers, is divided into five major subtypes based on histologic characteristics. The clinical characteristics, prognosis, and responses to treatments vary among subtypes. Here, we demonstrate that the variations of cell-cell contact energy result in the LUAD subtype-specific morphogenesis. We reproduced the morphologies of the papillary LUAD subtypes with the cellular Potts Model (CPM). Simulations and experimental validations revealed modifications of cell-cell contact energy changed the morphology from a papillary-like structure to micropapillary or solid subtype-like structures. Remarkably, differential gene expression analysis revealed subtype-specific expressions of genes relating to cell adhesion. Knockdown experiments of the micropapillary upregulated *ITGA11* gene resulted in the morphological changes of the spheroids produced from an LUAD cell line PC9. This work shows the consequences of gene mutations and gene expressions on patient prognosis through differences in tissue composing physical forces among LUAD subtypes.

## Highlights

• Cell-cell contact energy changed the tissue architecture of LUAD
• Differences in growth rate does not correlate to LUAD tissue architecture
• Differences in adhesion gene expressions causes LUAD tissue architecture
• Modification of the cell-cell contact energy changed the spheroid morphogenesis

## Graphical abstract

**Figure:**
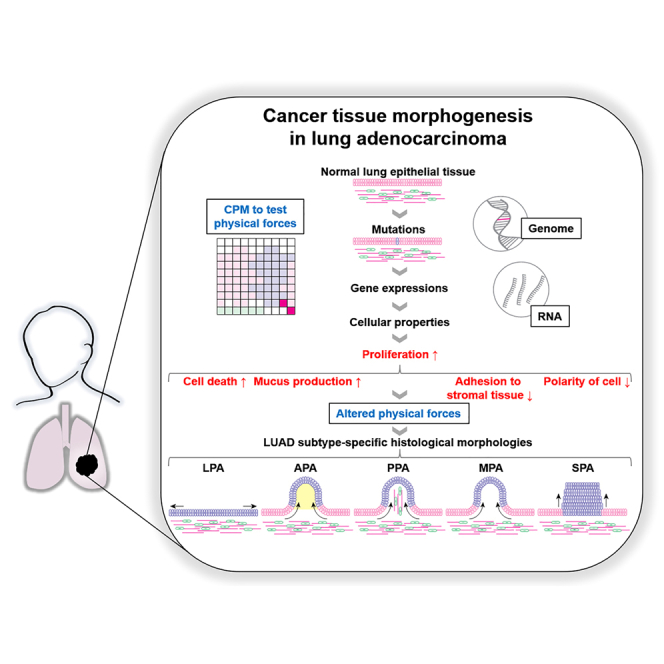

## Introduction

Despite recent improvements in treatment, lung cancer has been the leading cause of cancer deaths worldwide.^1^ Among them, non-small cell lung cancer (NSCLC) (WHO classification) accounts for most lung cancer cases, as determined by small biopsies and cytology samples for pattern typing, such as adenocarcinoma or squamous cell carcinoma.^2^ Lung adenocarcinoma (LUAD) represents about 40–50% of all lung cancers^3^^,^^4^ and is classified into five major subtypes with distinct characteristics: lepidic-predominant adenocarcinoma (LPA), acinar-predominant adenocarcinoma (APA), papillary-predominant adenocarcinoma (PPA), micropapillary-predominant adenocarcinoma (MPA), and solid-predominant adenocarcinoma (SPA). LPA is low-grade with a good prognosis; APA and PPA are intermediate grades; MPA and SPA are high-grade with a poor prognosis. In addition, MPA has high metastatic potential.^2^^,^^5^ The histological subtypes correlate with clinical courses, and each subtype has different responses to treatments. Thus, understanding the morphogenesis of each subtype should lead to a better understanding of subtype-specific characteristics, better predictions of clinical outcomes, and the development of better subtype-specific clinical treatments.

Several studies have reported comparisons of RNA expressions and genomic analysis of lung cancer tissues and showed that the LUAD subtypes can be classified into three groups based on the RNA expression and somatic genome signature analysis, including a transition-high subtype, a transversion-high subtype, and a structurally altered subtype.^6^^,^^7^^,^^8^ The three groups somewhat correlate with the histological subtypes and underlying mutations. However, it is difficult to predict the changes in tissue architectures of the LUAD subtypes by the classification from mass RNA expression data and genome analyses without knowing which part of the data we should focus on. Tissue architectures are emergent from the interactions among cells, surrounding tissues, and the local environment without requiring external directing influences.^9^ Tissue self-organization is a process where the overall architecture arises from local cell-cell and cell-matrix interactions. The proliferation of cells, cell deaths, cell sorting, and adhesions of each cell type produce pressure and surface tension leading to rearrangements. Such forces produce tissue patterns due to mechanical and spatial constraints. In normal tissues, the tissue architectures are maintained by balanced cell proliferation, differentiation, and death rates. Cancer tissue is also self-organized but driven by disturbed balances.^10^ The physical forces produced from these cellular properties directly control the tissue self-organizations, and the gene expressions controlling the forces are the points to which we should focus. Therefore, understanding how gene expression changes result in particular morphological changes requires understanding cell-cell interactions at the tissue level.

The cellular Potts model (CPM) is a multicell-scaled computational model that represents cells on a regular lattice and can describe complex cellular and multicellular behaviors.^11^^,^^12^ Simulations of the CPM incorporate mechanical forces and cell-cell interactions, which enable the CPM to reproduce the multicellular patterns of tissue development including cell sorting,^11^ cyst formation,^13^ tubular formation,^14^ and vascular network formation.^15^ We have used the CPM implementation in CompuCell3D (CC3D)^16^ to develop our models. CC3D is an open-source, cross-platform, CPM implementation that includes extensive control and visualization tools embedded in a user-friendly graphical user interface.

Here we develop CPMs that reproduce the tissue architectures of the five major LUAD subtypes. We started with PPA as the base model and showed that modifications of specific parameters resulted in the establishment of the other four subtypes. The hypotheses we used to reproduce the tissue architectures were validated by immunostaining of patient tissue sections, and the genes responsible for the tissue architectures were identified by gene expression studies across the LUAD subtypes. Our work shows the consequences of gene mutations and gene expressions on patient prognosis through differences in tissue composing physical forces among LUAD subtypes.

## Results

### Hypotheses to establish LUAD CPMs

**Figure 1:**
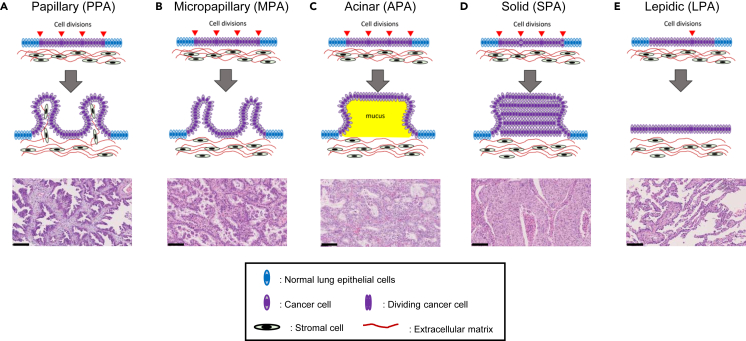
Schematic diagrams (upper) and HE stained sections (lower) of LUAD subtypes (A) The papillary subtype (PPA) has papillae filled with stromal tissue. (B) The micropapillary subtype (MPA) has papillae without stromal tissue. (C) The acinar subtype (APA) has papillae filled with mucus. (D) In the solid subtype (SPA), mass growth of cancer cells is observed with the epithelium becoming many cells thick. (E) In the lepidic subtype (LPA), cancer cells replace normal lung epithelial cells. Red triangles indicate cell division events and the number of triangles is the rate of division. Scale bars, 100 μm. See also Figure S1 and Table S1.

We initially set up hypotheses to implement in CPMs to reproduce the tissue architectures of the five LUAD subtypes (Figure 1). The lung epithelial layer is composed of a single layer of lung columnar epithelial cells, and LUAD is caused by the abnormal proliferation of cancerous columnar epithelial cells. The first morphology, PPA, is composed of papillae with stromal tissue. We hypothesized that PPA is caused by two processes: (1) Cell division causes the cancer epithelial layer lengthening and (2) increased tension force caused by the lengthening leads to protrusions of the epithelial layer (Figure 1A). These two hypotheses are a common basis for the tissue morphogenesis of the five LUAD subtypes, and we included additional hypotheses to reproduce the other four subtypes (see Table S1). The second morphology, MPA, is composed of smaller papillae without stromal tissue. In addition to the PPA hypotheses, we considered two additional possibilities for MPA: (1) Cell proliferation is too fast to produce protrusions that include stromal cells and matrix and (2) lower adhesion between cancer epithelial cells, stromal cells, and matrix resulted in epithelial protrusions without stromal cells and matrix (Figure 1B). The third morphology, APA, is composed of mucus-filled papillae. For APA, we hypothesize that cancer cells differentiate into secretory cells and secrete mucus in the papillae (Figure 1C). The fourth morphology, SPA, is composed of aggregations of cancer cells. We considered two possibilities: (1) High cell proliferation makes aggregates of cancer cells and (2) Cells are less differentiated and have low cell polarity, resulting in loss of epithelial characteristics and aggregated tissue architecture (Figure 1D). In the fifth and final morphology, LPA, cancer cells proliferate along the surface of intact alveolar walls. Our hypothesis for LPA is that the cell division rate is low and is almost the same as that of the normal lung epithelial cell death rate, resulting in the slow replacement of the normal lung epithelial cell layer with the cancer cell layer. The hypotheses for all five morphologies are summarized in Table S1.

### Tissue architecture reproduction of LUAD subtypes with CPMs

**Figure 2:**
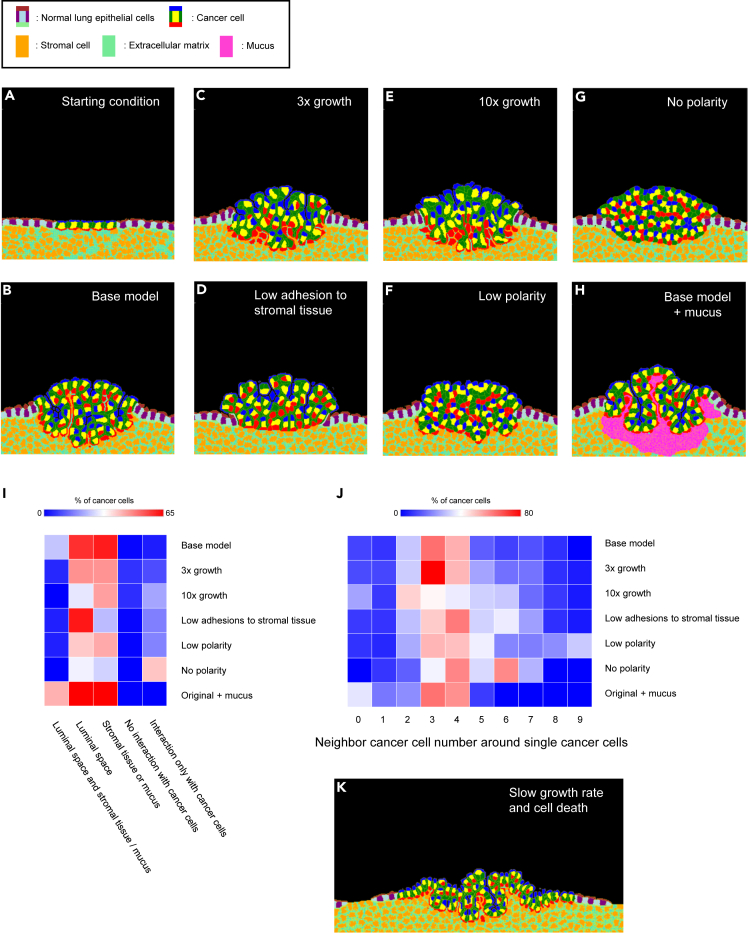
CPM simulations to reproduce LUAD subtype morphologies (A) The CC3D-CPM simulations started with 5 cancer cells placed in the center of a normal lung epithelial layer. Normal and cancer epithelial cells were composed of 4 subdomains: apical, basal, lateral, and cytosol. (B) In the base model, we set the growth rate of cancer cells as 0.03, the contact energy of tumor-basal to stromal cells and matrix as 3, the contact energy of tumor apical to other subdomains of tumor cells to 40, and the contact energy of tumor-basal to other subdomains of tumor cells to 40. The simulation was run for 4000 steps. (C) Cancer cell growth rate: 0.09. The simulation was run for 1400 steps. (D) Contact energy of tumor-basal to stromal cells and matrix: 30. The simulation was run for 4000 steps. (E) Cancer cell growth rate: 0.3. The simulation was run for 600 steps. (F) Contact energy of tumor-apical to other subdomains of tumor cells: 10. Contact energy of tumor-basal to other subdomains of tumor cells to 10. The simulation was run for 4000 steps. (G) The contact energy of each tumor subdomain was equally set as 10. The simulation was run for 4000 steps. (H) Mucus was added to the base model. The simulation was run for 4000 steps. (I) A heatmap shows how much percentage of cancer cells have specific types of connections: 1. Luminal space and stromal tissue/mucus, 2. Luminal space, 3. Stromal tissue or mucus, 4. No interaction with cancer cells, and 5. Interaction only with cancer cells. Averages of around 10 simulations per model are shown. (J) A heatmap shows how many the number of neighbor cancer cells around single cancer cells. Averages of around 10 simulations per model are shown. (K) Cell death was implemented for the reproduction of the lepidic subtype. Cancer cell death rate: 0.00065. Normal lung epithelial cell death rate: 0.0001. Cancer cell growth rate: 0.022. The simulation was run for 40000 steps. See also Figure S2, Tables S2, S3, and Video S1.

To reproduce the epithelial cell layer, compartmentalized cells were used for the cancer cells and the normal lung epithelial cells. These cells were composed of apical, basal, lateral, and cytosolic compartments in the model (inset of Figure 2). The normal and cancer virtual epithelial cell types have similar characteristics and are long columnar cells with lateral connections to adjacent cells to create the epithelial layer, apical membranes facing the luminal side, and basal membranes adhering to the stromal tissue (Figure 2A). In addition, the model included non-compartmentalized stromal cells, portions of extracellular matrix (ECM), and portions of mucus. For simplicity, the ECM and mucus portions were modeled as cells. The luminal space was filled with CompuCell3D “Medium”, which is a special cell type without volume or surface constraints.

The simulations started with five cancer cells in the lung epithelial layer. The proliferation of cancer cells lengthens the epithelial layer creating tension. The excess amount of epithelial layer was emergently folded, creating protrusions while retaining the connection to the underlying stromal tissue. Thus, a lengthened epithelial layer produced papillae with stromal tissue, which is the major characteristic of PPA (Figure 2B). We defined the PPA-reproducing CPM as the base model and modified the parameters of that base model to reproduce the other LUAD subtypes.

First, we increased the proliferation rate of the cancer cells. A 3-fold increase in the proliferation rate (3× growth) produced protrusions without stromal cell inclusions, which is similar to the characteristics of MPA (Figure 2C). The same result was produced by reduced adhesion of the cancer cells to the stromal tissue (low adhesions to stromal tissue) without modifying the proliferation rate from the base model (Figure 2D). The reduction of adhesion was reproduced by increasing the contact energy *j* value between the two tissue types. Since CPM tries to minimize system energy, increasing a contact energy *j* value results in decreased adhesion. A 10-fold increase in the proliferation rate (10× growth), or reduced epithelial polarity (low polarity), produced mass aggregations of cancer cells, which is characteristic of the SPA phenotype (Figures 2E–2G). The polarity control was also reproduced by modifying the contact energies. APA-like papillae filled with mucus were reproduced by the base model after the addition of mucus secretion by the cancer cells (Base model + mucus, Figure 2H). The parameters modified for the various models are given in Table S2, and the movie of the models is given in Video S1.

Video S1 Movies from CPMs, related to Figures 2 and 4 Movie demonstrating simulation results of CPMs with different parameter settings.

We scored the tissue architecture characteristics of each LUAD subtype by measuring the cell-cell interactions of each cancer cell with the following two criteria: (1) The types of neighbor cells and (2) The number of neighbor cancer cells (Figure S2). These scores allow the classification of the individual simulations as having produced one of the five LUAD phenotypes (Table S3). In the base and the base plus mucus models, higher percentages of cancer cells interact with both luminal and stromal tissue or mucus (Figure 2I). In the 10× growth and the low polarity models, higher percentages of cells interact with only cancer cells and with a higher number of cancer cells (Figures 2I and 2J). In the 3× growth and the low adhesions to stromal tissue models, most cells interact with luminal or stromal tissue, and the number of cancer cell-cancer cell interactions was intermediate (Figures 2I and 2J). We also show the relationship between the parameters and the cell-cell contacts in Figures S2H–S2J. Overall, faster growth rate, higher adhesion energy of cancer cells to stromal tissue, and lower polarity relate to mass aggregation of cancer cells (Figures S2H–S2J). Our hypotheses of tissue morphogenesis mechanisms successfully reproduced the distinct tissue architectures of four of the LUAD subtypes observed in clinical samples. However, we failed to reproduce the LPA tissue architectures with our hypotheses that the balanced rate of cancer cell proliferation and normal lung epithelial cell death leads to the LPA phenotype. With that hypothesis, cancer cells produced PPA-like papilla structures that contain protrusions partially filled with stromal tissue (Figure 2K).

### Higher cell death rates and stromal tissue proliferation are important to reproduce the tissue architecture of the LPA and the PPA, respectively

**Figure 3:**
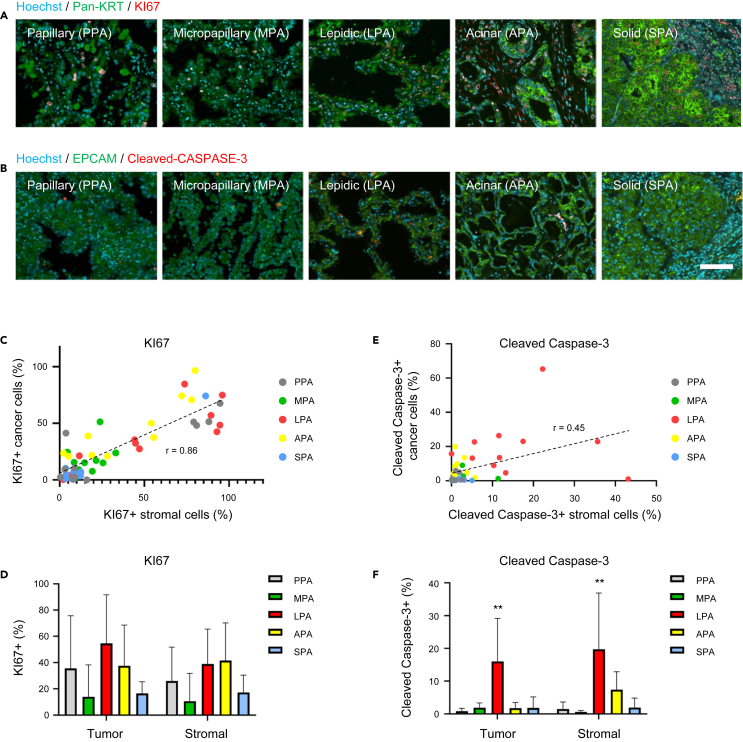
Patient tissue section analysis of proliferation and programmed cell death (A) Sections of the 5 LUAD subtypes stained with Hoechst (blue) and antibodies against pan-Keratin (green) and KI67 (red). (B) Sections of the 5 LUAD subtypes stained with Hoechst (blue) and antibodies against EPCAM (green) and cleaved-Caspase-3 (red). Scale bar: 100 μm. (C) The relationship between the KI67 positive cell ratio of cancer cells and stromal cells. The dotted regression line was fitted to all data. r: correlation coefficient. (D) KI67 positive cell ratio of cancer cells and stromal cells in the 5 subtypes. (E) The relationship between the cleaved-Caspase-3 positive cell ratio of cancer cells and stromal cells. The dotted regression line was fitted to all data. r: correlation coefficient. (F) KI67 positive cell ratio of cancer cells and stromal cells in the 5 subtypes. ∗∗*p* < 0.01, Tukey-Kramer. See also Figure S3.

We retrospectively collected tissue samples from patients with the five LUAD subtypes for immunofluorescence microscopy experiments. Tissue sections of each LUAD subtype were stained with epithelial cancer markers, pan-KERATIN and EpCAM, a proliferating cell marker, KI67, and an apoptotic cell death marker, cleaved-Caspase-3 (Figures 3A and 3B). We analyzed KI67 or cleaved-Caspase-3 positive cells in both cancer cells and cancer-related stromal cells. To measure the positive cell ratio in each cell type, we manually segmented the cancer cell layer and the stromal cell layer in the immunostained images (Figure S3). The segmented images were converted to binary data, and pixels were counted with ImageJ (Figure S3). Although we did not consider the proliferation of stromal cells in our models, we detected KI67-positive stromal cells in most of the tissue sections (Figures 3A and 3C). The ratio of KI67-positive stromal cells and cancer cells was well correlated (Figures 3C and 3D), suggesting stromal and cancer cells are often proliferating at comparable rates. In the models, we found the MPA and SPA subtypes were reproduced by the increased proliferation rate or the modifications of cell-cell contact energy for the adhesion/polarity reduction (Figures 2C–2F). On the other hand, we did not find significant differences in the ratio of KI67-positive proliferating cells among the five subtypes, suggesting that the proliferation rate is not important to the morphogenesis of the MPA and SPA subtypes (Figure 3D). Unlike KI67-positive cells, there was no correlation between the ratios of the cleaved-Caspase-3-positive dying cancer cells and stromal cells (Figure 3E). Interestingly, among the five subtypes, LPAs had significantly higher percentages of cleaved-Caspase-3-positive cells (Figure 3F). The ratio of KI67 or cleaved-Caspase-3-positive tumor and stromal cells are shown in Figures S3I and S3J.

**Figure 4:**
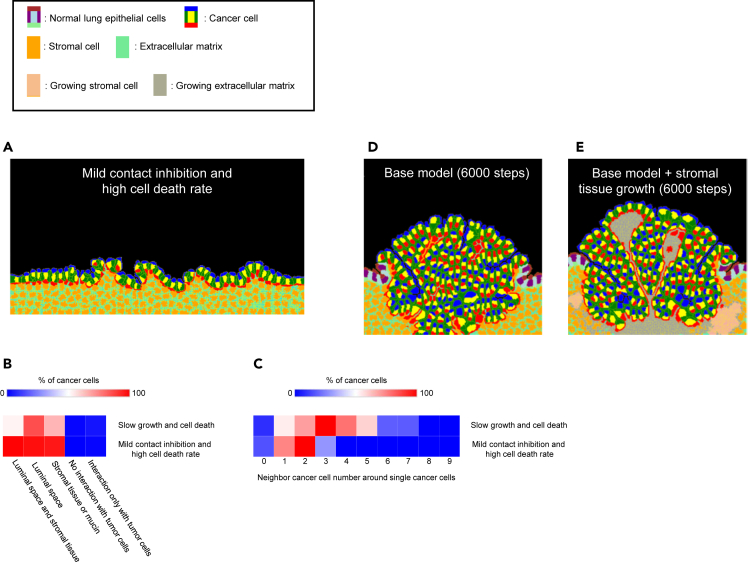
CPM simulations including conditions found in the immunostaining experiments (A) Lepidic-like morphology reproduction by the CPM with mild contact inhibition, cancer cell death rate: 0.002, normal lung epithelial cell death rate: 0.001, and cancer cell growth rate: 0.3. Contact inhibition was introduced with a lower growth rate when cancer cells contact other cells. The growth rate at contact inhibition was 99.7% of the set value. The simulation was run for 37500 steps. (B) A heatmap shows how much percentage of cancer cells have specific types of connections: 1. Luminal space and stromal tissue/mucus, 2. Luminal space, 3. Stromal tissue or mucus, 4. No interaction with cancer cells, and 5. Interaction only with cancer cells. Averages of 5 simulations per model are shown. (C) A heatmap shows how many the number of neighbor cancer cells around single cancer cells. Averages of 5 simulations per model are shown. (D) The base model was run for 6000 steps. (E) Growing stromal cells and extracellular matrix were implemented just beneath the cancer cells in the base model. The growth rate of the growing stromal cells and matrix was 0.02. The simulation was run for 6000 steps. See also Figure S4 and Video S1.

Next, we applied the conditions that we found in the immunostaining to the CPM simulations. We increased the cell death rate of cancer cells ten times with the addition of mild contact inhibition in the second LPA model. The contact inhibition was achieved by a 0.3% reduction of growth rate when the cancer cells attached to cancer cells or normal epithelial cells in the model. The second model produced almost a single cancer layer which replaced the normal lung epithelial cells (Figure 4A and Video S1). Compared to the initial LPA model, a higher percentage of cells faced both luminal space and stromal tissue indicating a lower production rate of papilla structures (Figures 4B, S4A, and S4B). The cancer cell-cancer cell interactions in the second model were smaller compared to the initial model (Figures 4C, S4A, and S4B). These characteristics are a good fit for the tissue architectures of the LPA subtype. The line plots shown in Figures S4C and S4D are the cellular number changes during the second LPA simulations. Compared to the initial LPA model, the timing of cell divisions was desynchronized because of the mild contact inhibition in the second LPA model (Figures S4C and S4D), which helped to reduce the sudden increase of tension force, caused by synchronized cell proliferation, and reduced production of PPA-like structures. Figure 4D shows a longer simulation result of the PPA model. Unlike the original shorter simulation result shown in Figure 2B, the longer simulation resulted in stromal cell-less protrusion formation (Figure 4D and Video S1). To account for this, we added a process of stromal cell proliferation, which was shown in our immunostaining experiments (Figure 3), to the PPA model. In the second model, papillae contained stromal tissue even in the longer simulations (Figure 4E).

### Differential gene expressions of LUAD subtypes correspond to the CPM predictions

**Figure 5:**
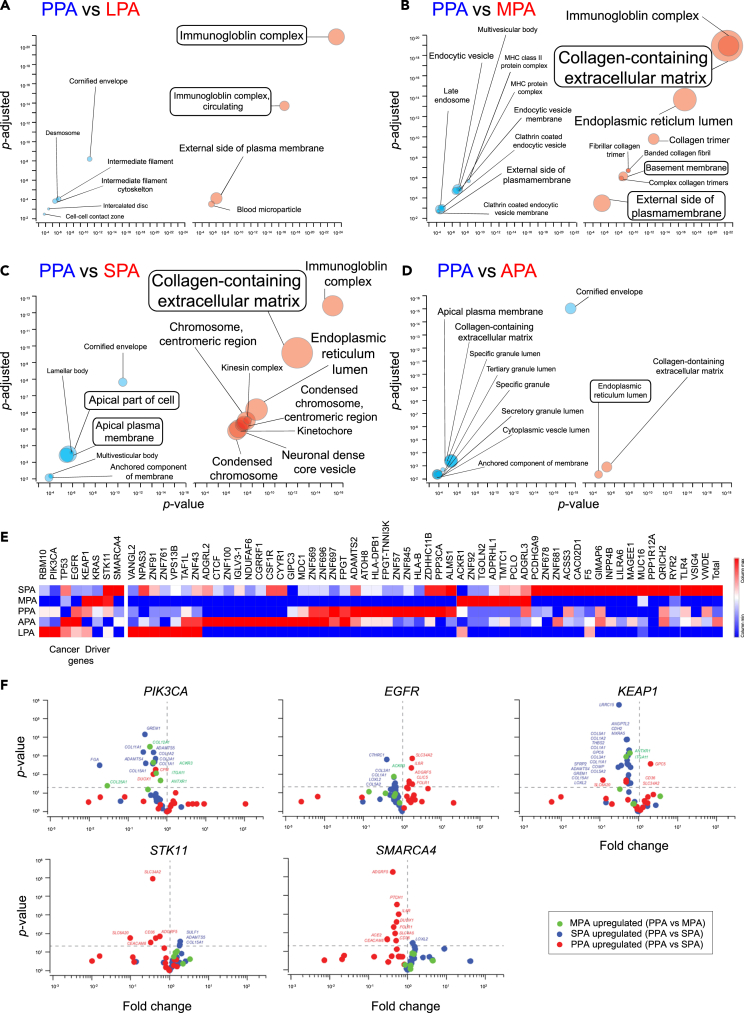
Differential gene expressions of the LUAD subtypes GO analysis of the papillary subtype (left panels) and the other subtypes (right panels) expressing genes. The sizes of the circles and texts are relative to the number of significant genes. The GO terms highlighted by boxes are the functions on which we focus. A maximum of the top 9 GO terms are shown. (A) GO analysis of the papillary subtype (PPA: blue circles in the left panel) and the lepidic subtype (LPA: red circles in the right panel) expressing genes. (B) GO analysis of the papillary subtype (PPA: blue circles in the left panel) and the micropapillary subtype (MPA: red circles in the right panel) expressing genes. (C) GO analysis of the papillary subtype (PPA: blue circles in the left panel) and the solid subtype (SPA: red circles in the right panel) expressing genes. (D) GO analysis of the papillary subtype (PPA: blue circles in the left panel) and the acinar subtype (SPA: red circles in the right panel) expressing genes. (E) Averaged mutation rates of each LUAD subtype. The heatmap of cancer driver genes and other genes is separated by a break. (F) Gene expression differences of MPA, SPA, and PPA upregulated genes listed in Table S5 dependent on the labeled mutations. Fold changes were calculated by the division of average FPKM values of samples with the labeled non-synonymous mutation and without non-synonymous mutation. Horizontal dashed lines show *p* = 0.05, Student’s *t* test. Vertical dashed lines show fold change = 1. See also Figure S5, Tables S4, and S5.

In our model simulations, we defined PPA as the base model, and modification of distinct parameters in the base model reproduced other LUAD subtypes. To validate this approach, we have compared gene expressions in PPA to the other LUAD subtypes. We used published LUAD gene expression and mutation data that contains 15 PPAs, 4 LPAs, 3 MPAs, 32 SPAs, and 27 APAs.^8^ The volcano plots were using differential gene expression data, and Gene Ontology (GO) analyses were performed with genes plotted with red dots in Figures S5A–S5D. In the comparison of PPAs and LPAs, we found LPAs express more genes in the immune system with Gene Ontology (GO) analyses^17^ (Figure 5A), which suggests higher immune response-related cancer cell deaths. Thus, the LPA-specific gene expression patterns may correspond to the observation of a high cell death rate of LPA in our immunofluorescent staining data (Figure 4A). For the reproduction of MPA and SPA, our model predicted that modifications of cell-cell contact energy resulted in low adhesion of cancer cells to the stromal tissue, and low polarity, respectively (Figures 2 and 3), and both properties relate to the cellular surface regulations. GO analyses showed that MPAs expressed more genes relating to extracellular and plasma membrane regulations compared to PPAs, like “collagen-containing ECM”, “basement membrane”, and “external side of the plasma membrane” (Figure 5B). SPAs also expressed more genes relating to extracellular and plasma membrane regulations than PPAs (Figure 5C). Compared to SPAs, PPAs expressed more genes relating to the apical plasma membrane (Figure 5C), suggesting that SPAs reduced apico-basolateral polarity compared to PPAs. This finding correlates well with our model prediction of low cell polarity in SPAs (Figures 2F and 2G). We found that APAs have higher gene expressions relating to the GO term of the endoplasmic lumen (Figure 5D). The GO term relates to the production of secretory proteins whose expressions were predicted in our model hypothesis of higher secretion in APAs (Figure 1C; Table S1).

Figure 5E shows the mutation rate of genes in each LUAD subtype. The mutation rates of cancer driver genes show some subtype specificity. Each subtype tends to have several specific mutations: LPAs with *RBM10* and *PIK3CA*, APAs with *TP53* and *EGFR*, PPA with *EGFR* and *KEAP1*, MPA with *KEAP1*, *STK11*, and *SMARCA*, and SPA with *TP53*, *STK11,* and *SMARCA4* mutations. The total mutation rates were high in APA and SPA subtypes, which have a higher rate of *TP53* mutation (Figure 5E; Table S4). We found some high mutation rate cell-cell adhesion-related genes including *ADGRL2* in APA, *TMTC1* and *ADGRL3* in MPA, and *PCDHGA9* in SPA subtypes (Figure 5E; Table S5). These mutations can directly participate in subtype-specific morphogenesis. In addition, we found high mutation rate transcription factor genes in each subtype (Figure 5E; Table S4). The mutation of transcription factors including chromatin remodeling proteins like *CTCF* and *SMARCA4* can control the subtype-specific morphogenesis through gene expression modifications. While we see the tendency of subtype-specific mutation patterns in the average data, each patient had different patterns of the combination of mutations, showing that the relationship between mutations and morphology is not simple (Figure S5E).

To understand the relationship between cancer-driver gene mutations and the LUAD subtype morphologies, we focused on genes that relate to cell-cell adhesion. From the GO classifications shown in Figures 5A–5D, we focused on the extracellular and plasma membrane regulatory classifications whose significance was validated by fold changes, *p*-values, and false discovery rates. We found 6, 30, and 17 candidate genes relating to MPA, SPA, and PPA morphologies, respectively (Table S5). We compared the expression levels of these 53 genes with or without each driver gene mutation. As shown in Figures 5F and S5F, the cancer driver gene mutations were mostly correlated with the reduction of the cell-cell adhesion gene expressions. Expression levels of MPA and SPA-upregulated genes were reduced in patients with *PIK3CA*, *EGFP*, or *KEAP1* mutations, and expression levels of MPA-upregulated genes were reduced in patients with *STK11* or *SMARCA4* mutations (Figure 5F). Especially, 5 of the 6 MPA-upregulated genes, 18 of the 30 SPA-upregulated genes, and 9 of 17 PPA-upregulated genes were downregulated in *PIK3CA*, *KEAP1*, and *SMARCA4* mutations, respectively, suggesting that the strong negative correlation of these subtype morphologies with the mutations (Figure 5F). In contrast, expression levels of some PPA-upregulated genes were increased in patients with *EGFP* mutations, and some outlier genes with increased expression in mutation samples were found (Figures 5F and S5F). These results suggest that the mutations of cancer driver genes control LUAD morphology through gene expression changes of cell-cell adhesion genes.

### Effects of subtype-specific expressing adhesion molecules in patient prognosis

**Figure 6:**
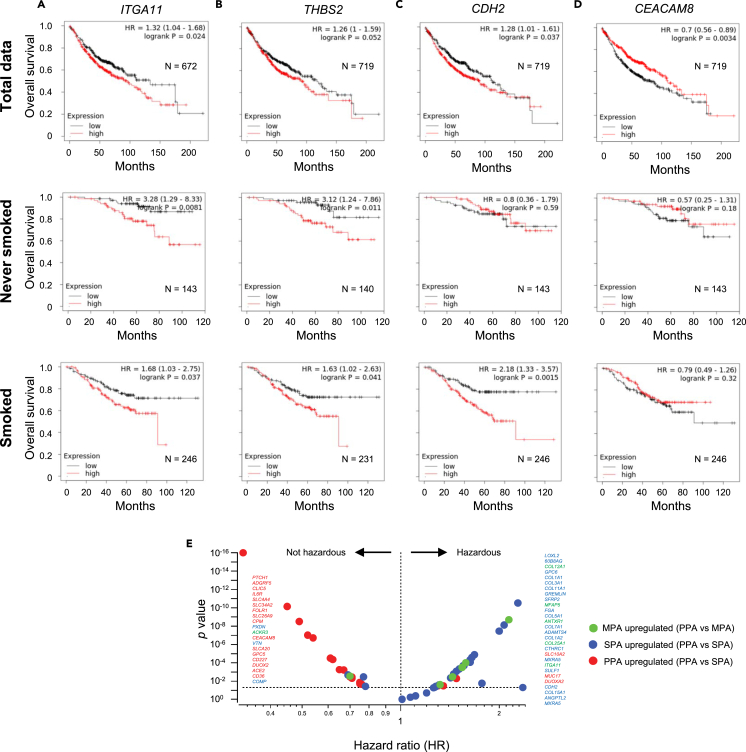
Survival analysis of differential genes among the LUAD subtypes (A) Survival plots evaluating the prognostic significance of the micropapillary upregulated *ITGA11* RNA expression in lung adenocarcinoma samples. (B) Survival plots evaluating the prognostic significance of the solid upregulated *THBS2* RNA expression in lung adenocarcinoma samples. (C) Survival plots evaluating the prognostic significance of the solid upregulated *CDH2* RNA expression in lung adenocarcinoma samples. (D) Survival plots evaluating the prognostic significance of the papillary upregulated *CEACAM8* RNA expression in lung adenocarcinoma samples. The top row panels are plots from total data. The middle and bottom row panels are plots of data from patients who never smoked and patients who smoked, respectively. Hazard ratios and *p* values by log rank test are shown in each panel (A-D). (E) Summary of the survival analyses of all differential genes listed in Table S4. The horizontal dashed line shows *p* = 0.05. The Vertical dashed line shows hazard ratio = 1. The significant gene names are listed in the panel. The order of the gene names is dependent on the *p* values. See also Figure S6 and Table S5.

From the morphology-related 53 genes listed in Table S4, we focused on one MPA, two SPA, and one PPA-related genes that directly affects cellular adhesion (Figure 6). MPA genes include *ITGA11,* which is not expressed in normal alveolar epithelial cells, is a receptor for collagen, and promoted tumorigenicity and metastasis in a study of NSCLC.^18^ We used the online database Kaplan-Meier plotter to investigate the relationship between gene expressions and patient prognosis.^19^ We found that *ITGA11* expression was significantly associated with the prognosis of LUAD patients in both individuals who smoked and those who never smoked (Figure 6A). SPA genes include *THBS2* and *CDH2*. THBS2 mediates cell-cell and cell-matrix interactions and is reported as a correlated factor of LUAD patients’ prognosis.^20^ Unlike the report, we did not find a significant association between *THBS2* expression and patient prognosis with total data (top panel of Figure 6B). However, significant associations were found between smoked and never-smoked datasets (middle and bottom panels of Figure 6B). N-Cadherin, coded by the *CDH2* gene, mediates cell-cell adhesion and has been reported as one of the genes important to LUAD prognosis.^21^ We found *CDH2* expression especially associated with smoked LUAD patient prognosis (Figure 6C). We also found that *CEACAM8* expression was high in the PPA subtype compared to the SPA subtype. CEACAM8, also known as CD66b, mediates cell adhesion and cell migration. We found low expression of *CEACAM8* associated with the LUAD patient prognosis (Figure 6D). We analyzed all differential genes listed in Table S5 (data from Figure S6), and Figure 6E and Table S5 show the summary of all differential gene results, showing that most of the PPA and MPA/SPA upregulated genes have positive and negative effects on patient survival, respectively (PPA: positive 84.2%, negative 15.8%, MPA: positive 20%, negative 80%, SPA: positive 10%, neutral: 16.7%, negative: 73.3%). We also explored the expressions of the morphology-related genes and clinical outcomes. We found 12 out of 19 PPA upregulated genes negatively 5 out of 6 MPA upregulated genes and 23 out of 30 SPA upregulated genes positively correlate to higher cancer grade (Figure S6E). We also found 5 PPA upregulated genes negatively and 3 MPA and 15 SPA upregulated genes positively correlated to metastasis (Figure S6E). We also show the relationships of the gene expressions and contralateral lung metastasis and liver metastasis and found the overall negative correlation to these factors in PPA genes (Figures S6F and S6G). We did not find any correlation between these genes and other clinical factors including metastasis to other organs and survival time.

### Spheroid morphology changes by adhesive gene knockdown

**Figure 7:**
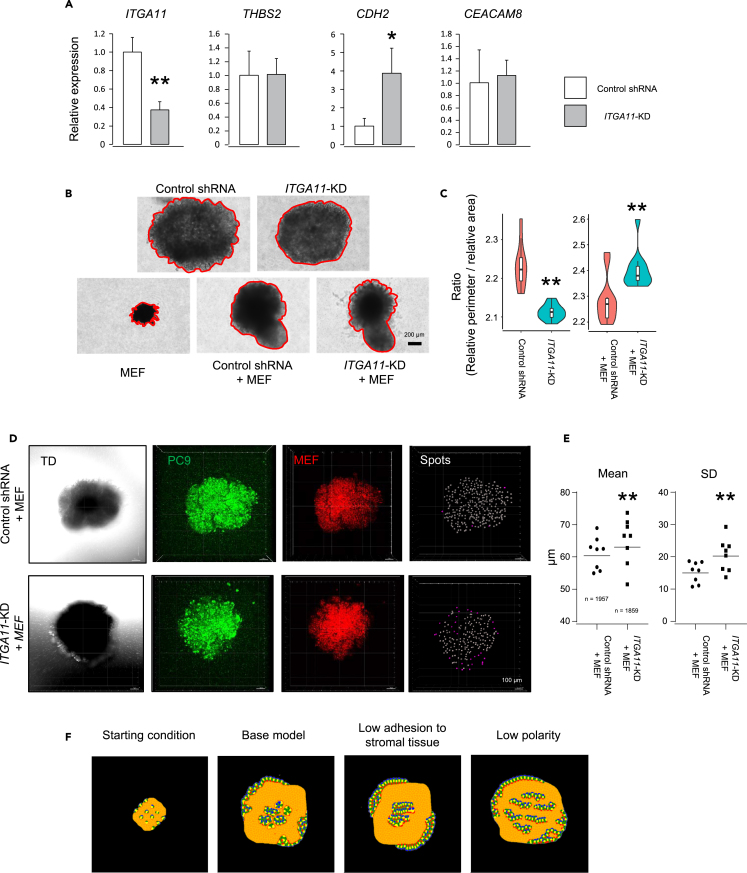
Adhesion molecule expression and spheroid morphology (A) Relative gene expression level of *ITGA11* in the stable PC9 cell lines of control-shRNA and shRNA against *ITGA11* (*ITGA11*-KD). (B) Morphological differences of spheroids produced from control-shRNA, *ITGA11*-KD, MEFs, a mixture of control-shRNA and MEFs, and a mixture of *ITGA11*-KD and MEFs. Red lines highlight the edge of each spheroid. Scale bar, 200 μm. (C) The structural differences of the spheroids. The ratio of the relative perimeter and the relative area were calculated for each spheroid and drawn as violin plots. ∗∗*p* < 0.01, students’ t-test. See also Figure S7. (D) 3D views of confocal images of mixture culture spheres of the PC9 cell lines and MEFs. TD means transmitted light differential interference contrast image. PC9 cell lines were stained with MitoTracker Green FM (ThermoFisher), and MEFs were stained with Track It Red (AAT Bioquest). Each MitoTracker labeled PC9 cell spot was detected by Imaris software (right panels). Magenta spots show cells where the distance to the 9 nearest neighbors is more than 60 μm. Scale bar, 100 μm. (E) The left and right graphs show the average and the standard deviation of distance to 9 nearest neighbors in each spheroid, respectively. Bars in the left and right graphs show the average and the standard deviation of distance to 9 nearest neighbors in all spots, respectively. ∗∗*p* < 0.01, students’ t-test and F-test for the average and the standard deviation, respectively. (F) Images obtained with CPMs. The simulations were started from the mixture of stromal cells and LUAD cells (left panel). CPM results of the “base model”, “low adhesion to stromal tissue”, and “low polarity” at 4000 steps are shown. See also Figure S7.

Our CPM models suggested that cellular adhesivity differences are important to the LUAD subtype-specific morphogenetic differences. The differentially expressed genes include genes that contribute to cell-cell interactions. To validate the function of differentially expressed genes in the LUAD morphogenesis, we performed shRNA knockdown (KD) of MPA-upregulated *ITGA11* in a lung cancer cell line PC9. PC9 is one of the most popular lung cancer cell lines and was established from an LUAD patient.^22^ However, there is no information about the histological subtype. Using these cells of unknown LUAD subtype, we selected stable transformant cell lines of control-shRNA and shRNA against *ITGA11*. *ITGA11*-KD cells not only have the reduction of *ITGA11* level (37.4 ± 0.1% of control-shRNA, Figure 7A) but also have higher *CDH2* expression (386.2 ± 1.4% of control-shRNA, Figure 7A). The high *CDH2* expression may compensate for the reduced cellular interaction in *ITGA11*-KD cells. *ITGA11* is one of the MPA-upregulated genes, and the *ITGA11*-KD cell line also has increased expression of the SPA-upregulated *CDH2* gene (Figure 7A). Thus, the *ITGA11*-KD cell line can be a model of PPAs or SPAs, and the control shRNA cell line can be a model of MPAs. We cultured the control and *ITGA11*-KD cells in the medium containing 2% methylcellulose (MC).^23^ In the MC media, cells do not have any scaffold and adhere to each other to produce spheroids (Figure 7B). Each type of cell shows distinctly shaped spheroids even though the culture started from the same number of cells (Figures 7B and S7A–S7E). The control-shRNA stable PC9 cells produced spheres with protrusions (Figures 7B and S7A). On the other hand, *ITGA11*-KD cells produced spheres without protrusions (Figures 7B and S7B), suggesting altered cellular adhesion compared to control-shRNA cells. The structural differences can be measured by the ratio of the perimeter and area of the spheroids. Since the perimeter is related to both the structural complexity and the area of spheroids (Figure S7F), we normalized the perimeter and the area of each spheroid (see STAR methods). The left graph in Figure 7C shows a significantly higher ratio of the relative perimeter vs. relative area of control-shRNA spheres compared to that of *ITGA11*-KD spheres due to complex morphology in the control-shRNA cells.

To check the stromal-cancer interaction, we cultured a mixture of the stable PC9 cell lines and stromal cells (mouse embryonic fibroblasts, MEFs). When the stable PC9 cell lines were cultured with MEFs in MC media, the mixed culture sphere was shaped like a mushroom, which consists of a cap and a stalk (lower panels in Figure 7B). The structures were slightly different between the two cell types: the cap of *ITGA11*-KD + MEF had more protrusions compared to that of control-shRNA + MEF (lower panels in Figures 7B, 7C, and S7C–S7E). The structural difference appeared to be a result of the cell-cell interaction difference between MEFs and the shRNA stable transformants of PC9. To visualize the distribution of both mixed cell lines, we pre-stained PC9 cell lines and MEFs before the start of spheroid cultures. The confocal microscope images show distinct distributions of PC9 cell lines (green cells in Figure 7D) in the mixed spheres. Compared to both cell lines, the control shRNA cells distributed more evenly than the *ITGA11*-KD cells in each spheroid (Figure 7D). The *ITGA11*-KD cell distribution was patchy and peripheral (lower columns of Figure 7D). We detected each green labeled PC9 cell with Imaris 3D image analysis software and measured the distances to the nearest neighbor cells. As shown in Figure 7E, we find the standard deviations of the distances were larger in the *ITGA11*-KD cells, suggesting the uneven distribution (*p* < 0.01, F-test, Figure 7E). To explain the difference in the distributions, we simulated the expected structures of mixed culture of the stromal cells and PPA, MPA, and SPA cells with CPMs of “base”, “low adhesion to stromal tissue”, and “low polarity”, respectively. The simulation was started from the even distribution of stromal cells and the LUAD cells (left panel of Figure 7F), and both cell types proliferate at the same rate. We simulated the three models for 4000 steps and found the SPA model (“low polarity”) produces a more even distribution of LUAD cells compared to the other two models (Figure 7F) in the simulated spheres. The sphere model’s morphological changes were not a simple model capable of producing all LUAD subtypes, but we show that the genetic alteration of one of the cell-cell adhesion molecules resulted in the changes of SPA-like distribution to PPA or MPA-like distribution.

## Discussion

Physical forces produced by cellular proliferation and cell-cell adhesion are the direct executors of tissue self-organization. In cancer cells, the forces are modified by altered gene expressions that are caused by sequential accumulation of somatic mutations. LUAD is classically divided into five subtypes based on the histomorphologic characteristics.^24^ The efforts to classify LUAD subtypes with RNA and genomic information resulted in the classification of three groups. The three groups show some sort of association with certain tissue types, but each subgroup contains a mixture of multiple tissue types.^6^^,^^7^^,^^8^ In this study, we analyzed the physical forces with CPMs, found parameters that need to be focused, searched genes related to the parameters, and found correlations between the specific gene expressions and the somatic mutations. With the strategy, we found clues of entire processes of cancer tissue morphogenesis in the five LUAD histological subtypes.

It is widely accepted that tumorigenesis is a multistep process that depends on a sequential accumulation of mutations within cells. The mutations result in increased cellular proliferation, decreased death, and changes in cell-cell adhesions and cell polarity. In our base CPM, the cancer cells have the same properties as normal epithelial cells except for an increased cellular proliferation. With properties of cellular polarity, interactions, and the spatial constraint of maintaining a surface, PPA-like papillae were emergent due to pressure and surface tension produced by cell proliferation (Figure 2B). In addition, we did not change epithelial properties in the models that reproduce APA and LPA morphologies (Table S2). Thus, the PPA, APA, and LPA cell properties are expected to be mostly the same as that of normal lung epithelial cells except for the increased proliferation, mucus secretion, and high cell death ratio, respectively. Note that the PPA, APA, and LPA patient prognoses are comparatively good among the five subtypes of LUAD.^5^

We initially assumed a higher proliferation rate is an important factor contributing to producing morphologies including MPA-like papillae which do not contain stromal tissues and SPA-like cancer cell aggregates. However, the staining of patient tissues showed varying KI67 positive proliferating cancer cell ratios in each LUAD subtype, and the average KI67 positive cell ratios were relatively low in the MPA and SPA subtypes (Figures 3C and 3D). This result was also supported by transcriptome analysis that did not show differential expression of proliferation-relating genes (Figure 5). This required us to change our focus from the proliferation rate to the strength of cell-cell adhesions for the reproduction of the morphologies of MPA and SPA. Cell-cell adhesion contributes to the polarity of cells and is generally reduced in cancer cells.^25^ Changes in the cellular adhesion alter the local mechanical forces, which results in modification of overall tissue architectures. In our CPM, changes in cell-cell contact energy resulted in cell-cell adhesions and cellular polarity differences that produced dynamic changes in PPA-like tissue structure, to MPA-like and SPA-like tissue structures (Figures 2D–2F and 2G).

Classically, the adhesion protein E-Cadherin was thought of as a central player of tumorigenesis because of the role of contact inhibition on cell proliferation in monolayer culture.^26^ However, recent 3D culture studies demonstrate that E-cadherin-mediated adhesion is not sufficient for conferring proliferation arrest.^27^^,^^28^ In comparison to the PPA subtype, we found differential expressions of 6 and 30 extracellular and plasma membrane-related genes that regulate cell adhesion and polarity in the MPA and the SPA subtypes, respectively (Figures 5D–5I; Table S5). Interestingly, these genes upregulated in MPA and SPA were mostly associated with a poor prognosis, and the genes upregulated in PPA were mostly related to a better prognosis (Figure S6; Table S5). These results suggest that the up and downregulated adhesion/polarity-related genes as a whole control pathological and tumorigenic differences between the LUAD subtypes.

We showed the knockdown of the MPA-specific gene, *ITGA11*, resulted in the morphological changes of spheroids shown in Figures 7 and S7. As we show in Figure 7A, the *ITGA11*-KD also resulted in the higher expression of the SPA-upregulated *CDH2* gene, suggesting further alterations of other adhesion gene expressions to compensate for the reduced adhesivity by the KD. ITGA11 promotes the adhesion of cells to the ECM, and N-cadherin, encoded in the *CDH2* gene, promotes homophilic adhesion of the expressing cells. Thus, the KD PC9 cells have higher homophilic adhesion and lower adhesion to MEFs in sphere culture (Figures 7D and 7E). The properties were reflected in the overall morphologies of the KD PC9 cell spheroids: packed smooth surface at single cultures and production of repulsive protrusions at mixture culture with MEFs (Figures 7B and 7C). As our expectation, the modification of the adhesion relating genes changed cell-cell contact and resulted in the difference in tissue self-organizations.

We are also curious about somatic mutations that produce gene expression changes promoting the subtype-specific tissue architecture changes of LUAD. Our mutational analysis also shows the subtype-specific enrichment patterns of the mutations of cancer driver genes and other genes (Figure 5E). Cellular proliferation and cell-cell adhesions work together to produce cancer (abnormal) morphologies. Most detected cancer driver mutations are related to cellular proliferation. Although some of the mutation genes are related to cell-cell adhesion, there is a gap between the accumulation of cancer driver mutations and subtype-specific gene expression changes of the cell-cell adhesion-related genes. Matano et al. reported engineered mutation induction of cancer driver genes into human colon organoids to produce tumors in transplanted mice.^29^ However, the driver mutation inductions alone cannot change the global gene expression and metastatic property of engineered organoids. In the mutations and expressions correlation analysis, we found negative correlations between cancer driver gene mutations and the subtype-specific cell-cell adhesion gene expressions (Figure 5F). We also found subtype-specific mutations in transcription factors and chromatin remodeling genes in the LUAD data (Figure 5E; Table S5), suggesting that chain reactions of transcription and epigenetic modifications would be necessary to promote the subtype-specific gene expression differences, and these processes may take a long time.

The negative correlation between the mutation and gene expressions can be useful to improve pathological methodologies based on artificial intelligence (AI). Developments in AI have enabled computational detection of tumors in hematoxylin and eosin (HE)-stained slide imaging, which is called computational pathology. Comparing the tumor detection rates, AI achieved a 92.4% sensitivity while a pathologist achieved 73.2%.^30^ Recently, Fujii et al. reported that detections of *BRAF* and microsatellite instability mutations of colorectal cancer were achieved by the computational pathology from HE-stained patient slides.^31^ The correlation of morphology and cancer driver mutations in LUAD were also reported.^32^ AI perhaps could identify the mutations and morphological relationships, but it is difficult to interpret what features the AI is focusing on and how the differences are produced. The negative correlations of the cancer driver mutations and the subtype-specific cell-cell adhesion gene expressions are one of the insights into the morphological different appearances that could be incorporated to improve the accuracy of the computational pathology. Not only the pathological improvements, but the findings of negative and positive metastasis-related genes (Figures S6E–S6G) also provide us with potential clinical targets to prevent metastasis of LUAD cancer cells in the patients.

The sequential accumulation of mutations amplifies the alteration of cellular properties including proliferation, cell death, adhesion, and polarity. The cellular properties, especially adhesion and polarity, affect the mechanical forces that are important for tissue morphogenesis. These forces have both a direct effect on tissue morphology as well as indirect effects through mechanosensory receptors and other feedback mechanisms. The mechanosensory receptors alter gene expression through signal transduction or stretching of chromatin creating additional changes to the factors contributing to the morphology of the tissue.^33^^,^^34^ Thus, the study of cancer tissue architecture requires understanding the orchestrated effects of the immediate effects of changes in processes like adhesion, as well as the feedback-mediated mechanisms giving rise to additional changes in the critical morphological controlling process.

We have collected clues on several aspects of LUAD including cellular forces, gene expression changes, and cellular morphology. CPM simulations and experimental validations revealed two basic sources of LUAD morphogenesis: tension forces produced by cellular proliferation and cell-cell contact energy. The tension force works as a common morphogenetic source, and the differences in cell-cell contact energy produce subtype-specific morphologies. In addition, the unique excess rate of cell death produces the outlier LPA morphology. The combination of hypothesis-driven computational modeling, with purpose-driven experimentation for refinement and validation, highlights the power of the simultaneous use of both approaches. Approaches such as this are critical to increase our mechanistic understanding of normal and abnormal tissue morphologies. Increased mechanism-based understanding is likely to lead to better clinical interventions.

### Limitation of the study

As with all computational models, only objects and processes included in the model can affect model outputs. Therefore, results are limited by our understanding of the key characteristics of the biological system. We have used bulk tissue RNA data to help define the model and this assumes a level of cellular homogeneity that may be incorrect. In the future, single-cell RNA data may help expand our understanding of cell-to-cell variability and allow more precise definition of cell behaviors. Alternatively, in the absence of single-cell RNA data, our model could incorporate small changes in the parameters for individual cells. For example, different cell-cell adhesions or cell proliferation rates (chosen from a distribution about our best estimate of optimal values) for each cell to establish if model outputs are critically dependent on homogeneous cells, or if cell-to-cell variability is tolerated.

In addition, we have not exhaustively explored parameter space and hence have not produced or examined all possible tissue morphologies that the base model might produce. To this end, we are exploring clustering methods to better understand the output morphologies. We are exploring both traditional clustering using user-defined descriptors of final cell layouts, such as the neighbor counts used in this study (see Figures 2I and 2J), as well as unsupervised machine learning to cluster the simulation output images into morphologically distinct cell layout classes. Great strides have been made in image clustering using machine learning techniques and, to our knowledge, those techniques have not been applied to the analysis of computationally produced tissue images such as those shown in Figures 2A–2H.

## STAR★Methods

### Key resources table

**Table:** 

REAGENT or RESOURCE	SOURCE	IDENTIFIER
**Antibodies**		

Anti-Pan-Keratin	Cell Signaling Technology	Cat#4545; RRID: AB_490860
Anti-Cleaved-Caspase-3 (Asp175)	Cell Signaling Technology	Cat#9661; RRID: AB_2341188
Anti-EpCAM (MOC-31)	Thermo Fisher	Cat#MA5-12442; RRID: AB_10982453

**Chemicals, peptides, and recombinant proteins**		

Hoechst 33342	Thermo Fisher	H1399
Mayer’s Hematoxylin Solution	Sakura finetek	6187–2
Methylcellulose	Sigma-Aldrich	M0512
PBS	NISSUI	63-5753-70
RPMI1640	FUJIFILM Wako pure chemical	189–02025
Trypsin EDTA solution	FUJIFILM Wako pure chemical	209–16941
Polyethylene glycol 6000	Sigma-Aldrich	1546580
Puromycin	InvivoGen	ant-pr-1
MitoTracker Green FM	ThermoFisher	M7514
Cell Explorer Live Cell Tracking Kit Red Fluorescence	AAT Bioquest	22623

**Critical commercial assays**		

RNeasy Mini Kit	Qiagen	74104
Fast SYBR Green Master Mix	Thermo Fisher	4385610
EndoFectin lenti	GeneCopoeia	EF001

**Deposited data**		

Experimental data	This paper	Database: https://data.mendeley.com/drafts/xc4392398v
The source code for all the CC3D models	This paper	Database: https://hdl.handle.net/2022/29394.
RNA seq and mutation data of LUAD subtypes	Soltis et al., 2022^8^	https://gdc.cancer.gov/about-data/publications/APOLLO-LUAD-2022

**Experimental models: Cell lines**		

Human: PC9 cells	RIKEN BRC	RCB4455

**Oligonucleotides**		

Primer: *TBP* Forward: TGTGCACAGGAGCCAAGAGT	This paper	N/A
Primer: *TBP* Reverse: ATTTTCTTGCTGCCAGTCTGG	This paper	N/A
Primer: *ITGA11* Forward: CTGCACACCGGACCCAG	This paper	N/A
Primer: *ITGA11* Reverse: CCCACGACCAGCCACTTATT	This paper	N/A
Primer: *THBS2* Forward: GGAGGACTTGGACGGTGATG	This paper	N/A
Primer: *THBS2* Reverse: CCTTTGGGATCCAAGGGGAC	This paper	N/A
Primer: *CDH2* Forward: AAGGACAGCCTCTTCTCAATGT	This paper	N/A
Primer: *CDH2* Reverse: CTTCTGCTGACTCCTTCACTG	This paper	N/A
Primer: *CEACAM8* Forward: GTTCAGCGTACATCCGGAGA	This paper	N/A
Primer: *CEACAM8* Reverse: GGGTGACTGGGTCACTGAAG	This paper	N/A
shRNA target sequence: scramble shRNA: CCTAAGGTTAAGTCGCCCTCG	Sarbassov et al.^38^	https://www.addgene.org/1864/
shRNA target sequence: *ITGA11*: GCCATCCAAGATCAACATCTT	Sigma-Aldrich	https://portals.broadinstitute.org/gpp/public/clone/details?cloneId=TRCN0000057755

**RecombinantDNA**		

psPAX2	Addgene	Plasmid #12260
pCMV-VSVG	RIKEN BRC	RDB04392
scramble shRNA plasmid	Addgene	Plasmid #1864
MISSION shRNA plasmid for *ITGA11*	Sigma-Aldrich	TRCN0000057757

**Software and algorithms**		

CompuCell3D Versions 4.3.2 and 4.4.1	Swat et al., 2012^16^	https://compucell3d.org/
ImageJ (Fiji)	NIH	https://fiji.sc/
Spectral Unmixing Plugin	Zimmermann et al., 2002^35^	https://imagej.nih.gov/ij/plugins/spectral-unmixing.html
R	R Foundation	https://www.r-project.org/
Igor Pro 4.04	WaveMetrics	https://www.wavemetrics.com/
Illustrator CS 5.1	Adobe	https://www.adobe.com/products/illustrator.html
edgeR	Robinson et al., 2010^36^	https://bioconductor.org/packages/release/bioc/html/edgeR.html
Enhanced Volcano	Bligh et al., 2018^37^	https://github.com/kevinblighe/EnhancedVolcano
ClusterProfiler	Wu et al., 2021^17^	https://bioconductor.org/packages/release/bioc/html/clusterProfiler.html
Morpheus	Broad institute	https://software.broadinstitute.org/morpheus
Kaplan-Meier Plotter	Gyorffy et al., 2013^19^	https://kmplot.com/analysis/
intOgen	Martinac et al., 2020^33^	https://www.intogen.org/search
Imaris 10.0	Oxford instruments	https://imaris.oxinst.com/support/imaris-release-notes/10-0-0

### Resource availability

#### Lead contact

Further information and requests for resources should be directed to and will be fulfilled by the lead contact, Toru Matsu-ura (matsutor@hirakata.kmu.ac.jp).

#### Materials availability

This study did not generate new, unique reagents.

#### Data and code availability

Data reported in this paper will be shared by the lead contact upon request. The source code for the CPMs generated in this study are available at https://hdl.handle.net/2022/29394. Any additional information required to reanalyze the data reported in this paper is available from the lead contact upon request.

### Experimental model and study participant details

#### Case and data selection

55 cases of non-small cell lung cancer data from Japanese patients who underwent lung cancer resection at Kansai Medical University Hospital between 2009 and 2015 were extracted from our institutional database for retrospective chart review and used in this study. That is, completely resected pTNM (pathological stages (pStages)) pathologically with a pathological diagnosis of NSCLC. In this paper, the main study was conducted using samples of lung adenocarcinomas, which were classified into the following histopathological types: lepidic (LPA), acinar (APA), papillary (PPA), micropapillary (MPA), and solid (SPA) adenocarcinomas. 11 cases for each subtype, 30 males, 25 females, and ages from 52 to 86 patients’ data were used. Distributions of ages for each subtype are not significantly different. The average and the standard deviation of ages for each subtype are 69.73 ± 7.73 for LPA, 67.64 ± 10.35 for APA, 69.00 ± 8.00 for PPA, 65.45 ± 7.31 for MPA, and 67.53 ± 8.06 for SPA.

### Method details

#### Cellular potts model

For our simulations we used CompuCell3D (CC3D) software (https://compucell3d.org/).^16^ CC3D is an implementation of the Cellular Pots Model (CPM) that simulates cells as sets of pixels in a 2D or 3D lattice. To reproduce the apical – basal – lateral morphology of the epithelial cell layer, compartmentalized cells were used for both the cancer and normal lung epithelial cells. These two cell types consisted of apical, basal, lateral, and cytosolic sub-compartments in the model (inset of Figure 2A). The two virtual epithelial cell types have similar characteristics and are columnar epithelial layer cells. They have lateral adhesions to adjacent epithelial cells, apical membranes facing the luminal side, and basal membranes connecting to the stromal tissue (Figure 2A). In addition, the model included non-compartmentalized stromal cells, and portions of extracellular matrix and mucin, which were modeled as small pseudo-cells. The luminal space was filled with CC3D “medium”, which is a special cell type that lacks volume constraints. Cell compartments and compartment aggregates (“compartmental cells”) and regular non-compartmental, cells included target volume and energy scaling factor parameters along with definitions of adhesion energies both within compartment cells (e.g., between the cytosolic and basal sub-compartments) as well as adhesions between different cells. Note that no surface constraints were used on any of the cells, cell compartments or pseudo-cells.

The models are two dimensional (2D) and were run on a square lattice measuring 500 by 500 pixels. A lattice pixel represents approximately 1 μm and cells were typically about 10x10 pixels, though the exact sizes change for different cell types and state during model execution. The columnar epithelial cells were nominally 10 pixels wide and 30 pixels tall. The CPM “neighbor order” was three, meaning each pixel functionally has 12 neighbor pixels over which effects can be felt. The CPM algorithm is an energy trajectory that samples a time course of plausible configuration and is not a pure energy minimizer. The range of acceptable configuration energies is controlled by a Bohltzman Distribution using a “Potts Temperature”. This “temperature” controls acceptable ranges of energies that the simulation can access. A Potts “temperature” of 10 was used for most of our simulations. The Simulations were run for between 5000 and 50,000 Monty Carlo Steps (MCS) with each MCS representing approximately 2 h of simulated time. Therefore, simulations typically represented a time course in the range of two months to one year. Our key time calibration is a 24-h mitosis cycle for cell division. Beyond that basic timescale, we accelerated times somewhat to make simulations more practical. Since the CPM is a stochastic model, we ran 10 replicate simulations for each parameter set. Metrics, such as cell counts, were averaged over the replicates.

The source code for all the CC3D models is available as a ZIP archive from https://hdl.handle.net/2022/29394.

The cell types, parameters, and variables used in the simulations are available in the source code files and are summarized in the Supplemental Tables listed below.

Table S1: Hypothesized emergence of LUAD phenotypes due to changing cell behaviors. +: upregulated, ++: highly upregulated, and -: same as normal epithelial cells.

Table S2: General CC3D-CPM characteristics and simulation parameters in each model. NA means not applicable.

Table S3: Differences of cell-cell interaction modes relate to the morphological phenotypes.

Table S6: Capabilities of the cell types in the CPM simulations.

Heat maps for the quantified CPM results were created with the online tool Morpheus (https://software.broadinstitute.org/morpheus).

#### Fluorescent immunostaining analysis

2 mm diameter circular tissue columns were extracted from the paraffin blocks of the 55 LUAD tissues and embedded into single blocks to produce tissue microarrays (TMAs). Each TMA contains one tonsil tissue and 11 tissues from one of the LUAD subtypes. Sections from TMAs were stained with primary, and fluorescent labeled secondary antibodies. We used the following dye and antibodies: Hoechst 33342 (Thermo Fisher, #H1399), anti-Pan-Keratin (Cell Signaling, #4545), cleaved-Caspase-3 (Asp175) (Cell Signaling, #9661), and EpCAM (MOC-31) (Invitrogen, #MA5-12442). Fluorescent images were obtained through an upright microscope (BX63; Olympus) with a cooled charge-coupled device (CCD) camera (ORCA-R^2^; Hamamatsu Photonics) and a 10x, 0.40 NA, objective (Olympus). Obtained images were analyzed with Spectral Unmixing Plugin installed in ImageJ.^35^

#### Lung adenocarcinoma cell culture and knockdown (KD) experiments

We cultured PC9 lung adenocarcinoma cells with RPMI1640 media supplemented with 10% fetal bovine serum, penicillin, and streptomycin (PC9 culture media). We used lentivirus vectors to express shRNAs. Lentivirus vectors were produced by transfections of packaging plasmids and a transfer plasmid with EndoFectin lenti (GeneCopoeia, #EF001) transfection reagent into Hek293TA packaging cells (GeneCopoeia). Hek293TA cells were cultured with DMEM supplemented with 10% fetal bovine serum, penicillin, and streptomycin. Lentivirus containing medium was harvested after 48 h of transfection and concentrated with 8% polyethylene glycol. Infected PC9 cells were selected with 1 μg/mL puromycin (InvivoGen, ant-pr-1). We used the following packaging vectors and transfer vectors: psPAX2 (Addgene, Plasmid #12260), pCMV-VSV-G (RIKEN BRC, RDB04392), scramble shRNA (Addgene, Plasmid #1864),^38^ and shRNA expressing transfer plasmid (Sigma-Aldrich, MISSION shRNA) for *ITGA11* (TRCN0000057757).

#### Materials

psPAX2 was a gift from Didier Trono (Addgene plasmid # 12260; http://n2t.net/addgene:12260; RRID:Addgene_12260). The pCMV-VSV-G was provided by the RIKEN BRC through the National BioResource Project of the MEXT, Japan (cat. RDB04392).

#### Spheroid culture and analysis

Cell lines were cultured in 2% methylcellulose (MC) containing PC9 culture media. MC media was produced as described.^23^ Briefly, 2 g MC (M0512; Sigma-Aldrich) was bottled with stirrer bar and autoclaved. 100 mL PC9 culture media was applied in the bottle, and the solution was stirred overnight to dissolve the MC in a refrigerator (4°C). Cells were suspended in the culture media at the concentration of 0.5 × 10^5^ cells/μL. Then, 1 μL each droplet was added into the MC media, and cultured for one week. Each droplet grew as a spheroid. The bright field images were obtained with an inverted microscope (CKX53; Olympus) with a CCD camera (DP23M; Olympus), a 4×, and a 10× objectives (Olympus).

Pre-staining of the PC9 cell lines and MEFs were performed before starting of the mixture cultures. Suspended single cells of PC9 cell lines and MEFs were placed in separate 1.5 mL tubes and incubated with 1 μM MitoTracker (ThermoFisher) and 1× Track It Red (Cell Explorer Live Cell Tracking Kit Red Fluorescence, AAT Bioquest), respectively, in a CO_2_ incubator at 37°C for 1 h. The stained cells were mixed at 1:1 ratio and cultured for one week in MC media. The fluorescent images were obtained with FV3000 scanning confocal microscope (Olympus) with a 10× objective (Olympus), a 488, and a 561 lasers (Olympus).

The perimeter and area of each spheroid were analyzed with ImageJ. The structural differences can be measured by the ratio of perimeter and area of the spheroids. Since the perimeter is not only related to the structural complexity but also the size of spheres, we normalized the perimeter (P) and the area (A) of each spheroid. Assuming the shape of each spheroid is a circle, we estimated the radiuses (R) from the perimeter and the area separately. The equation for the radiuses from the perimeter (Rp) and the area (Ra) are:

$$Rp=\frac{P}{2\pi },Ra=\sqrt{A/\pi }$$

The two radiuses (Rp and Ra) estimated separately from P and A were divided by Ra to calculate normalized radiuses (nRp and nRa). We calculated the normalized perimeter (nP) and the normalized area (nA) with the two different normalized radiuses.

$$nP=2\pi Rp,nA=\pi {Ra}^{2}$$

The ratio of nP and nA (nP/nA) is used as the index of the structural complexity of each spheroid.

### Quantification and statistical analysis

#### Analysis of differential gene expressions and mutations among the LUAD subtypes

We used RNA expression data of the LUAD subtypes reported by Soltis et al.,^8^ which contains sequencing data of RNA extracted from 4 lepidic, 27 acinar, 15 papillary, 4 micropapillary, and 32 solid predominant adenocarcinoma tissues. The data was downloaded from dbGap study accession #phs003011.v1.p1, from the NCI Cancer Research Data Commons at https://gdc.cancer.gov/about-data/publications/APOLLO-LUAD-2022.

To find the differentially expressed genes between each combination of the LUAD subtypes, we performed likelihood ratio tests using the edgeR R package.^36^ The differential gene data were used to draw volcano plots with the Enhanced Volcano R package.^37^ Gene ontology (GO) analyses for differentially expressed genes were calculated with the ClusterProfiler R package.^17^ Differential genes with *p*-values smaller than 0.05 were analyzed. We used the Cellular Component sub-ontology dataset for the analyses. In Figure 5, the GO terms were replotted against the *p*-values and *p*-adjusted values with Igor Pro 4.04 (WaveMetrics). The graphs were edited by Illustrator CS 5.1 (Adobe) to modify the size of each circular marker and add the annotation of GO terms. The sizes of circular markers and the annotations were relative to the number of the significant genes in each GO term.

We used mutation data from whole-genome sequencing of each LUAD patient reported by Soltis et al.^8^ Mutational cancer driver genes were detected from the list of mutation genes with online tool intOgen.^33^ Heat maps for the somatic synonymous mutation results were created with the online tool Morpheus (https://software.broadinstitute.org/morpheus).

#### Survival analysis

The correlations between the differential genes and survival in the LUAD patients were determined with Kaplan-Meier Plotter.^19^ We used gene chip analyzed mRNA expression of the differential genes from a total of 1162 LUAD patients. We also analyzed patients’ smoking histories.

#### Mutation analysis

We used data reported by Soltis et al.^8^ Gene expressions data of 53 genes listed in Table S5 was divided to two groups dependent on the existence of non-synonymous mutation in each cancer driver gene. Expression levels (FPKM values) of the each two group were compared with Student’s t test. Average FPKM value of non-synonymous mutation group was divided by that of no mutation/synonymous mutation group to calculate fold change for each gene.

#### 3D analysis of fluorescent stained spheroids and statistical analysis

3D spheroid images obtained by FV3000 scanning microscope (Olympus) were analyzed by Imaris 10.0 software. Each fluorescent spot was detected, and average distance to 9 nearest neighbors of each spot was measured. The software output the mean and standard deviation (SD) of the distance for all spots in single spheroid. We analyzed 8 spheroids for each experimental condition.

The total mean value was calculated from total sum of distances and total number of spots. The total SD value was calculated from total sum of squared deviation and total number of spots. Students’ t-test was performed by the calculation of the statistic amount from the total number of spots, the total averages, and the total SD values, and the statistic amount was compared to studentized range list. F-test was performed by the calculation of the statistic amount from the total sum of squared deviation and the total number of spots, and the statistic amount was compared to F-distribution list.

## Supplemental information

Document S1. Figures S1–S7 and Tables S1–S6

Figure S1. ▪▪▪

Figure S2. ▪▪▪

Figure S3. ▪▪▪

Figure S4. ▪▪▪

Figure S5. ▪▪▪

Figure S6. ▪▪▪

Figure S7. ▪▪▪
