# Supplementary material for: Morphological basis of the lung adenocarcinoma subtypes
Source: iScience. 2024 Apr 12;27(5):109742. doi: 10.1016/j.isci.2024.109742 (PMC11066476; doi:10.1016/j.isci.2024.109742)
Supplement: Document S1. Figures S1–S7 and Tables S1–S6 [file mmc1.pdf]

iScience, Volume *27*

Supplemental information

Morphological basis of the lung adenocarcinoma subtypes

Linjun Zha, Toru Matsu-ura, James P. Sluka, Tomohiro Murakawa, and Koji Tsuta

Supplemental figures

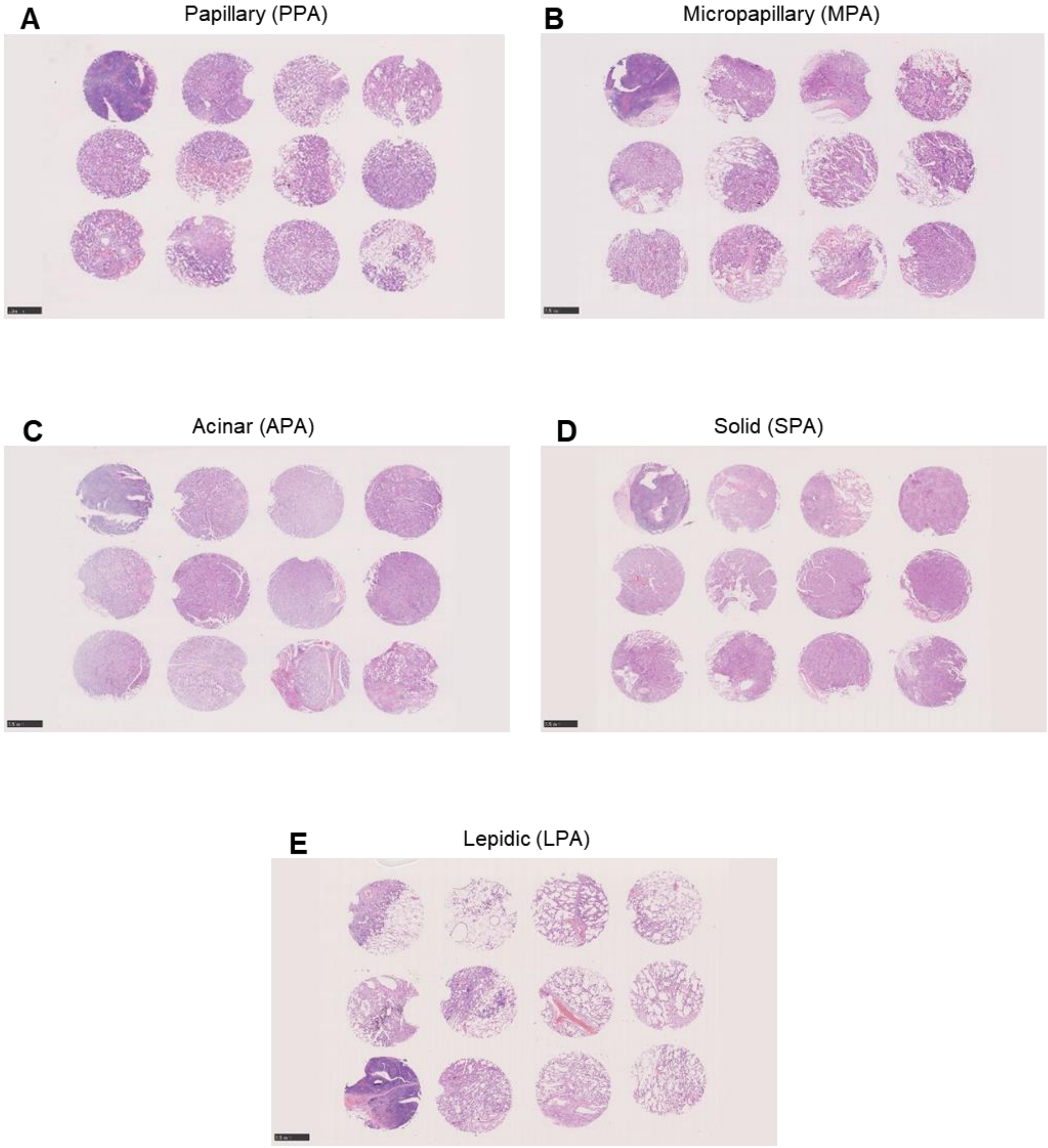

Figures S1: HE staining of each LUAD subtype. Related to Figure 1. HE staining of tissue microarrays of papillary (**A**), micropapillary (**B**), acinar (**C**), solid (**D**), and lepidic (**E**) subtypes. The microarrays located at the left-top in **A**-**D** and the left-bottom in **E** are control-tonsil tissue. Scale bars, 2.5 mm.

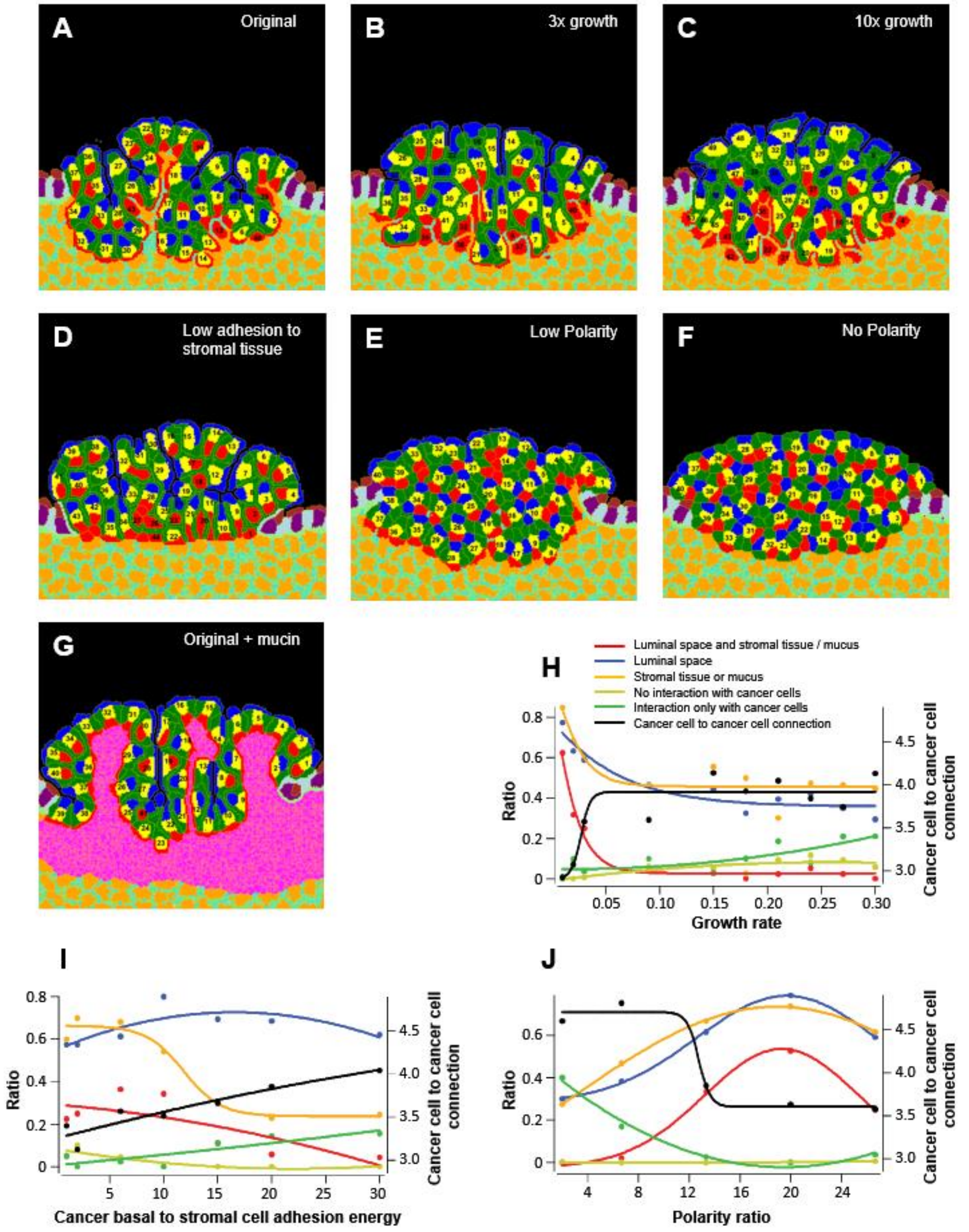

Figures S2. Cell-cell interaction analysis of CC3D-CPM simulations. Related to Figure 2. Cancer cells were numbered in each simulation of the models: base CC3D-CPM (**A**), three-time growth rate (**B**), ten-time growth rate (**C**), one-tenth adhesion to stromal tissue (**D**), one-fourth polarity (**E**), one-tenth polarity (**F**), and base plus mucin (**G**). Connecting cell types and numbers were manually counted for each cancer cell. (**H-J**) How much percentage of cancer cells have

cell-cell connections of each criterion and how many the number of neighbor cancer cells around single cancer cells are plotted against growth rate (**H**), the cancer basal to stromal cell adhesion energy (**I**), and the polarity ratio (**J**). The polarity ratio is calculated by the ratio of the highest j value to the lowest j value in the compartments of cancer cells.

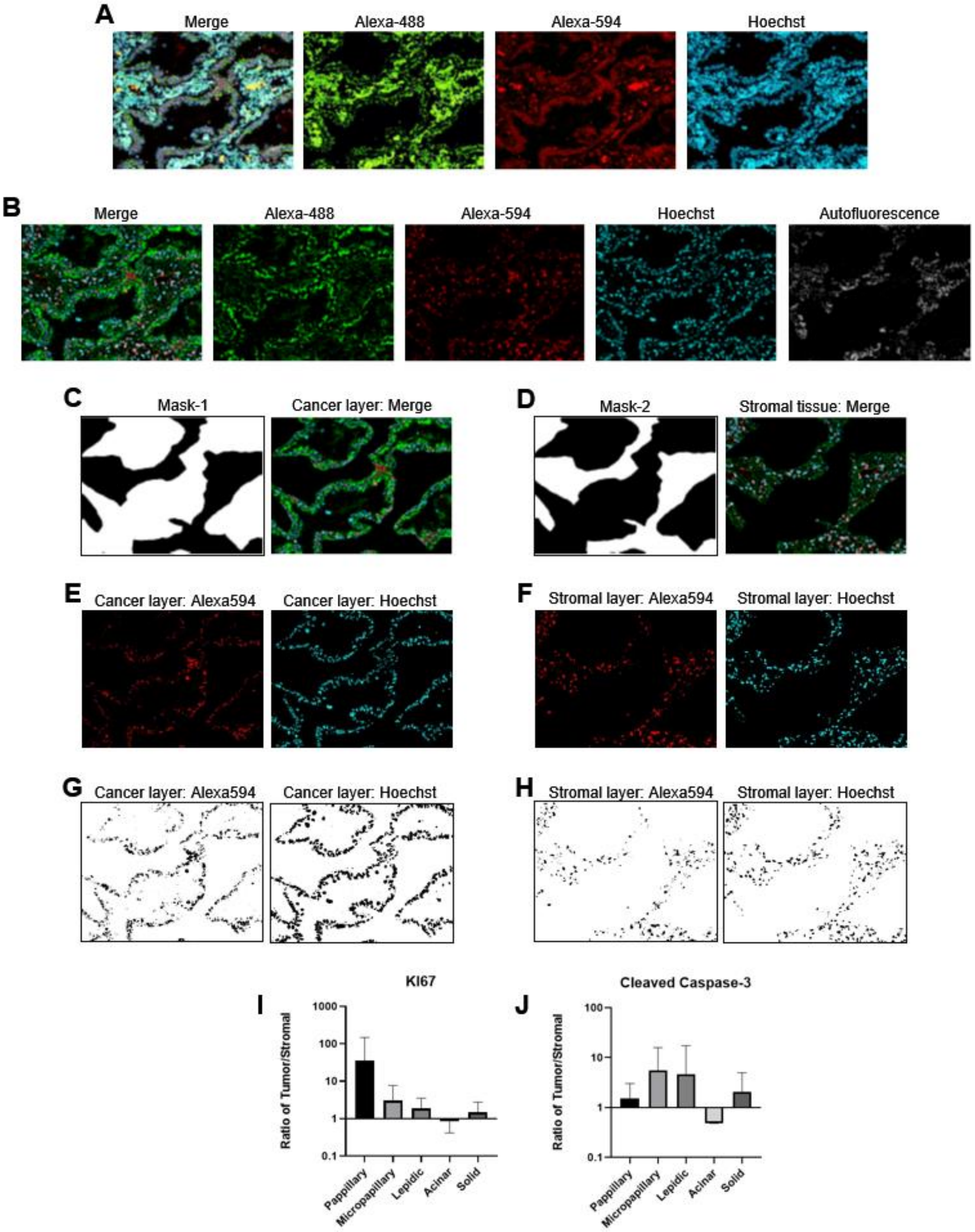

Figures S3. Image processing of fluorescent immunostainings. Related to Figure 3. Tissue sections were stained with three fluorescent immunostains: Alexa-488, Alexa-594, and Hoechst. The original images (A) were subjected to fluorescent unmixing to eliminate autofluorescence (B). We manually drew two masks (C and D) to separate the cancer layer and stromal tissues by using cancer marker pan-Keratin or EpCAM staining images. E and F show the separated cancer layer (E) and stromal tissue (F) images, respectively. The separated images were binarized and pixels

were counted to calculate the ratio of staining cells. (**I**) The ratio of KI67 positive tumor cells and KI67 positive stromal cells in the 5 subtypes. (**J**) The ratio of cleaved Caspase-3 positive tumor cells and cleaved Caspase-3 positive stromal cells in the 5 subtypes.

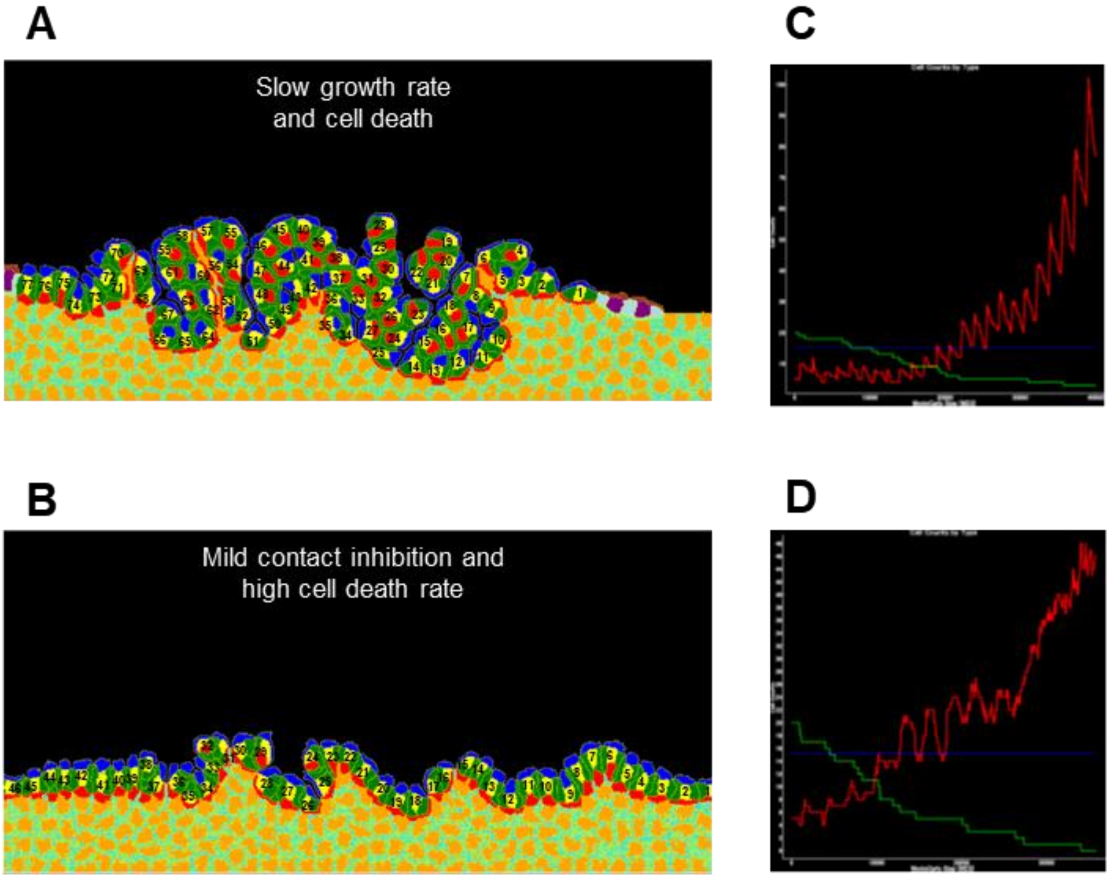

Figures S4. Cell-cell interaction analysis of the lepidic type CPM simulations. Related to Figure 4. Cancer cells were numbered in each simulation. Results for the models: slow growth rate and cell death (**A**), and mild contact inhibition, and high cell death rate (**B**). Connecting cell types and numbers were manually counted for each cancer cell. The number of epithelial cells was plotted in each model: slow growth rate and cell death (**C**), mild contact inhibition, and high cell death rate (**D**). Red, green, and blue lines represent cancer, normal lung epithelial cells, and stromal, respectively. The vertical axis is the number of each cell type. The horizontal axis is the simulation step.

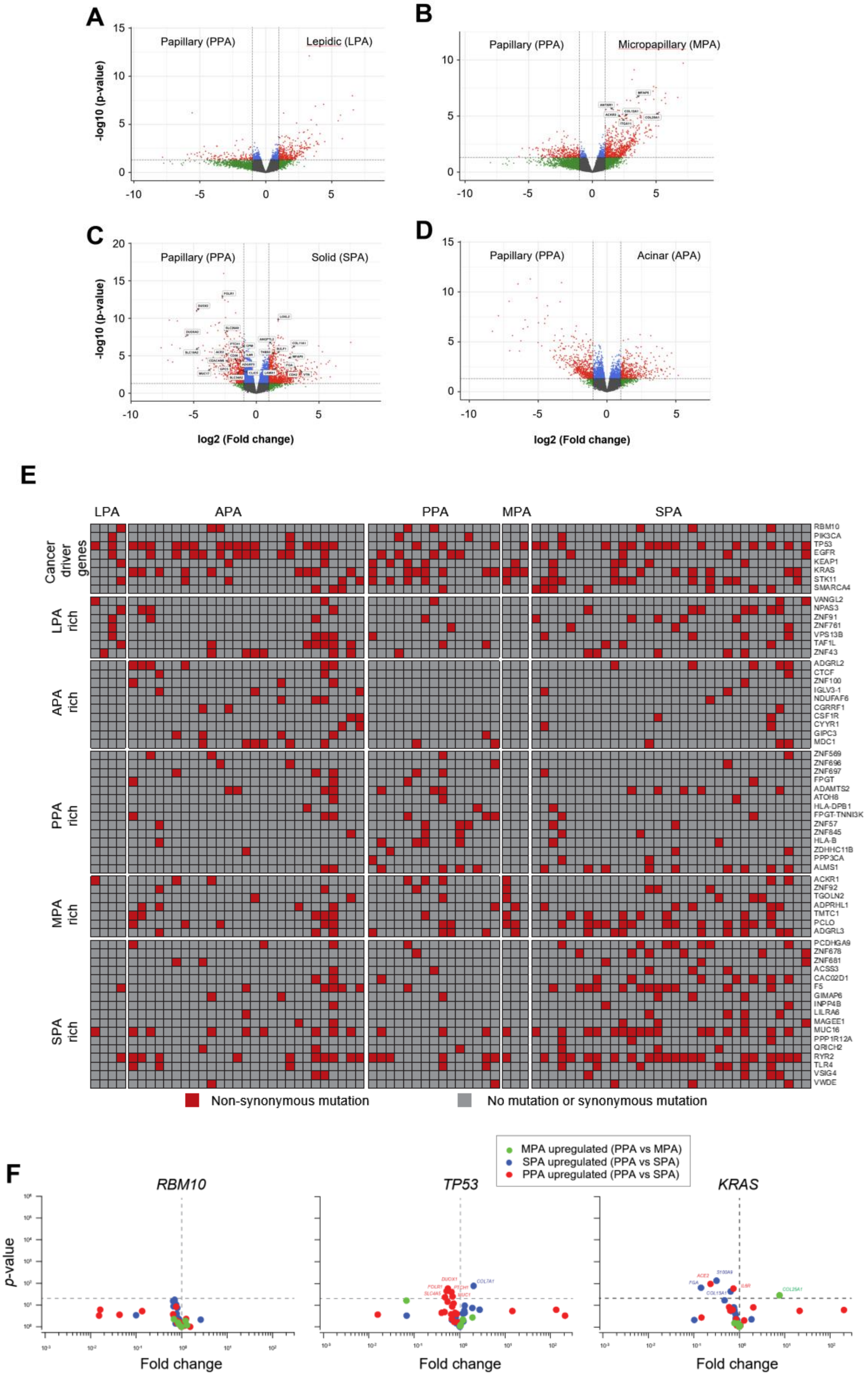
A
Papillary (PPA)
Lepidic (LPA)
B
Papillary (PPA)
Micropapillary (MPA)
C
Papillary (PPA)
Solid (SPA)
D
Papillary (PPA)
Acinar (APA)
-log10 (p-value)
log2 (Fold change)
E
LPA
APA
PPA
MPA
SPA
Cancer driver genes
LPA rich
APA rich
PPA rich
MPA rich
SPA rich
RBM10
PIK3CA
TP53
EGFR
KEAP1
KRAS
STK11
SMARCA4
VANGL2
NPAS3
ZNF91
ZNF761
VPS13B
TAF1L
ZNF43
ADGRL2
CTCF
ZNF100
IGLV3-1
NDUFAF6
CGRRF1
CSF1R
CYYR1
GIPC3
MDC1
ZNF569
ZNF696
ZNF697
FPGT
ADAMTS2
ATOH8
HLA-DPB1
FPGT-TNNI3K
ZNF57
ZNF845
HLA-B
ZDHHC11B
PPP3CA
ALMS1
ACKR1
ZNF92
TGOLN2
ADPRHL1
TMTC1
PCLO
ADGRL3
PCDHGA9
ZNF678
ZNF681
ACSS3
CACO2D1
F5
GIMAP6
INPP4B
LILRA6
MAGEE1
MUC16
PPP1R12A
QRICH2
RYR2
TLR4
VSIG4
VWDE
Non-synonymous mutation
No mutation or synonymous mutation
F
MPA upregulated (PPA vs MPA)
SPA upregulated (PPA vs SPA)
PPA upregulated (PPA vs SPA)
RBM10
TP53
KRAS
p-value
Fold change

Figures S5. Differential gene expressions of the LUAD subtypes. Related to Figure 5. (**A-C**) Differential gene expression analysis of the papillary versus the lepidic subtypes. A volcano plot of PPA against LPA (**A**), MPA (**B**), SPA (**C**), and APA (**D**). Vertical dotted lines show ± 1 of log2 fold change. Horizontal dotted lines show $p = 0.05$. (**E**) Mutations of each LUAD sample. Columns are each LUAD patient sample, and rows are genes with mutation. The heat map is divided by breaks for LUAD subtypes and types of gene mutations. (**F**) Gene expression differences of MPA, SPA, and PPA upregulated genes listed in Table S5 dependent on the labeled mutation. Fold changes were calculated by the division of average FPKM values of samples with the labeled non-synonymous mutation and those without non-synonymous mutation. Horizontal dashed lines show $p = 0.05$, Student's t-test. Vertical dashed lines show fold change = 1.

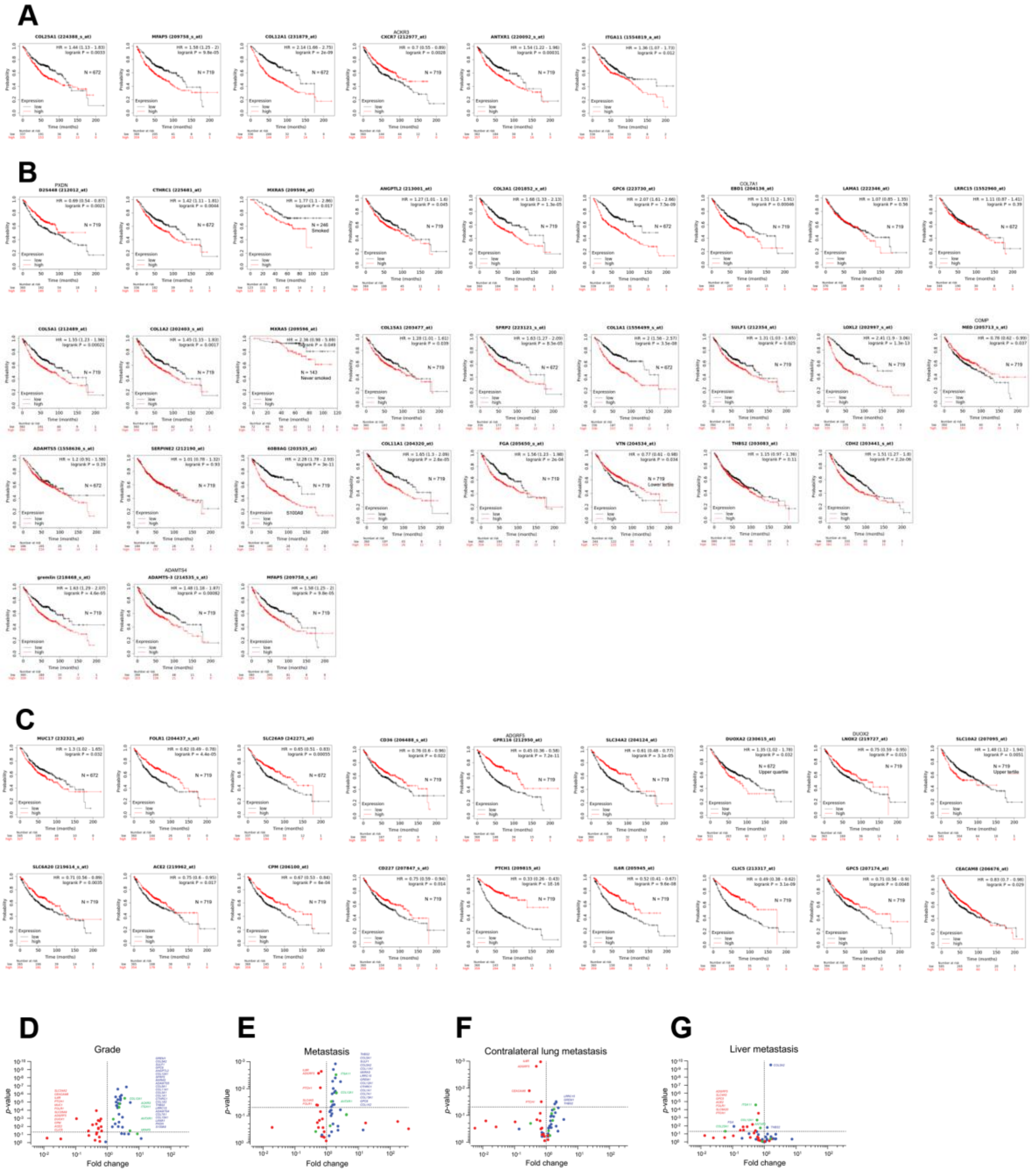

Figures S6. Survival analysis of differential genes among the LUAD subtypes. Related to Figure 6. Survival plots to evaluate the prognostic significance of upregulated genes in each LUAD subtype are shown. The results of differential genes upregulated in the micropapillary (**A**) or the solid (**B**) subtypes against the papillary subtype are shown. (**C**) The results of differential genes upregulated in the papillary subtype against the solid subtype are shown. The sub-panel numbers 1, 2, and 3 represent total, never smoked, and smoked patient populations, respectively. (**D-G**) Gene expression differences of MPA, SPA, and PPA upregulated genes listed in Table S5 dependent on the labeled clinical conditions. Horizontal dashed lines show p = 0.05, Student's t-

test. Vertical dashed lines show fold change = 1. (**D**) Grade means cancer grade, and the data contains LUAD patients with grade 2 or 3 stages. The fold change was calculated by the division of average FPKM values of samples with grade 3 patients and those with grade 2 patients. (**E-G**) The fold change was calculated by the division of average FPKM values of samples with the labeled clinical conditions and those without the conditions.

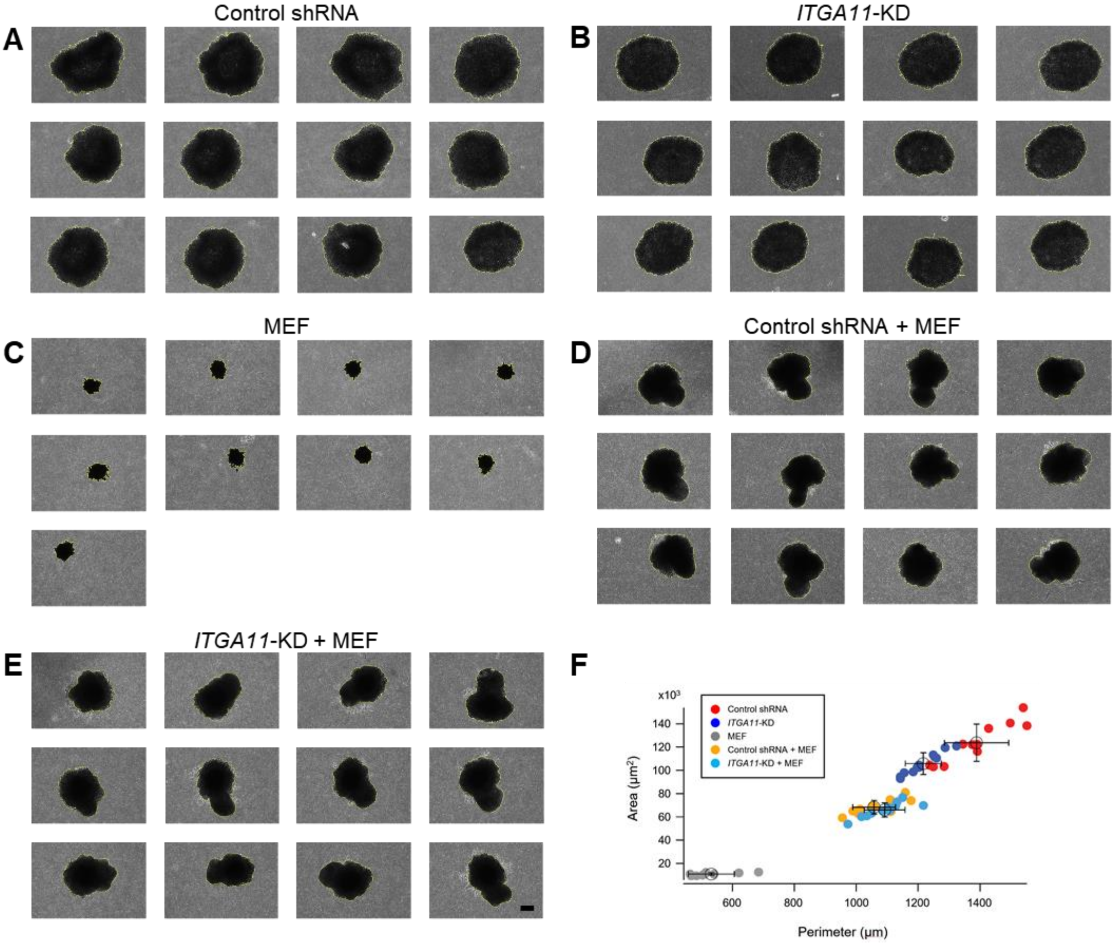

Figures S7. Morphological differences of stable PC9 cell lines and the mixture with MEFs. Related to Figure 7. Morphologies of PC9 cells expressing control-shRNA (**A**), *ITGA11*-targeted (*ITGA11*-KD) (**B**), MEFs (**C**), a mixture of control-shRNA and MEFs, and a mixture of *ITGA11*-KD and MEFs (**D**). Scale bar, 200 μm. (**F**) Distributions of area (μm^2) and perimeter (μm) of each spheroid. Opened circle with bars, mean ± SD.

LUAD Type:	PPA	MPA	APA	SPA	LPA
**Observations:→<br>Mechanism: ↓**	**Stroma filled papillae**	**Empty papillae**	**Mucus filled papillae**	**Cancer cell aggregation**	**Static epithelial length**
Cancer epithelial proliferation	+	++	+	++	-
Cancer epithelial tension	++	++	+	+	-
Cancer to stromal and matrix adhesion	-	+	-	-	-
Cancer cells differentiate into secretory cells	no	no	yes	no	no
Cancer cells loose polarity	no	no	no	yes	no

Table S1 Hypothesized emergence of LUAD phenotypes due to changing cell behaviors, related to Figure 1.
+: upregulated, ++: highly upregulated, and -: same as normal epithelial cells.
LUAD: lung adenocarcinoma, PPA: papillary-predominant adenocarcinoma, MPA: micropapillary-predominant adenocarcinoma, APA: acinar-predominant adenocarcinoma, SPA: solid-predominant adenocarcinoma, LPA: lepidic-predominant adenocarcinoma

Model Characteristic	CPM*[1] time step (approx)	Total simulated time (approx)	CPM pixel width (approx)	CPM lattice size pixels	CPM neighbor order
Value	2 hours	1 year (Base model: 4000 steps)	1 μm	500 x 500 x 1 (2D)	3

Models	Coefficients for growth and cell death				Contact energy *j*					
	Growth rate (pixels/mcs*[2])	Cancer cell death rate (% chance of death/mcs)	Normal epithelial cell death rate (% chance of death/mcs)	Contact inhibition	Tumor-basal to stromal cells and matrix	Tumor-apical to tumor-apical	Tumor-apical to other subdomains of tumor cells	Tumor-lateral to tumor-lateral	Tumor-lateral to other subdomains of tumor cells	Tumor-basal to other subdomains of tumor cells
Base	0.03	NA	NA	NA	3	80	40	5	40	40
3x growth	0.09	NA	NA	NA	3	80	40	5	40	40
10x growth	0.3	NA	NA	NA	3	80	40	5	40	40
Low adhesions to stromal tissue	0.03	NA	NA	NA	30	80	40	5	40	40
Low polarity	0.03	NA	NA	NA	3	20	10	5	10	10
No polarity	0.03	NA	NA	NA	3	10	10	10	10	10
Original + mucus	0.03	NA	NA	NA	3	80	40	5	40	40
Slow growth + cell death	0.022	0.065	0.01	NA	3	80	40	5	40	40
Mild contact inhibition + high cell death rate	0.03	0.2	0.1	0.97	3	80	40	5	40	40

***j* values in each model ↓**

**Models: Base, 3x growth, 10x growth, Slow groth rate and cell death, Mild contact inhibition and high cell death rate**											
Contact energy*[3]	Medium	Tumor_apical	Tumor_lateral	Tumor_basal	Tumor_cytosol	Epi_apical	Epi_lateral	Epi_basal	Epi_cytosol	Stromal	Matrix
Medium	0	4	30	30	30	4	30	30	30	30	30
Tumor_apical	4	80	40	40	40	80	40	40	40	40	40
Tumor_lateral	30	40	5	40	40	40	5	40	40	40	40
Tumor_basal	30	40	40	40	40	40	40	40	40	3	3
Tumor_cytosol	30	40	40	40	40	40	40	40	40	40	40
Epi_apical	4	80	40	40	40	80	40	40	40	40	40
Epi_lateral	30	40	5	40	40	40	5	40	40	40	40
Epi_basal	30	40	40	40	40	40	40	40	40	3	3
Epi_cytosol	30	40	40	40	40	40	40	40	40	40	40
Stromal	30	40	40	3	40	40	40	3	40	3	1
Matrix	30	40	40	3	40	40	40	3	40	1	1
Internal adhesion energy*[4]	Medium	Tumor_apical	Tumor_lateral	Tumor_basal	Tumor_cytosol	Epi_apical	Epi_lateral	Epi_basal	Epi_cytosol	Stromal	Matrix
Tumor_apical	-	5	5	20	5	-	-	-	-	-	-
Tumor_lateral	-	5	5	5	5	-	-	-	-	-	-
Tumor_basal	-	20	5	5	5	-	-	-	-	-	-
Tumor_cytosol	-	5	5	5	5	-	-	-	-	-	-
Epi_apical	-	-	-	-	-	5	5	20	5	-	-
Epi_lateral	-	-	-	-	-	5	5	5	5	-	-
Epi_basal	-	-	-	-	-	20	5	5	5	-	-
Epi_cytosol	-	-	-	-	-	5	5	5	5	-	-
**Model: Low adhesion to stromal tissue**											
Contact energy	Medium	Tumor_apical	Tumor_lateral	Tumor_basal	Tumor_cytosol	Epi_apical	Epi_lateral	Epi_basal	Epi_cytosol	Stromal	Matrix
Medium	0	4	30	30	30	4	30	30	30	30	30
Tumor_apical	4	80	40	40	40	80	40	40	40	40	40
Tumor_lateral	30	40	5	40	40	40	5	40	40	40	40
Tumor_basal	30	40	40	40	40	40	40	40	40	30	30
Tumor_cytosol	30	40	40	40	40	40	40	40	40	40	40
Epi_apical	4	80	40	40	40	80	40	40	40	40	40
Epi_lateral	30	40	5	40	40	40	5	40	40	40	40
Epi_basal	30	40	40	40	40	40	40	40	40	3	3
Epi_cytosol	30	40	40	40	40	40	40	40	40	40	40
Stromal	30	40	40	3	40	40	40	3	40	3	1
Matrix	30	40	40	3	40	40	40	3	40	1	1
Internal adhesion energy	Medium	Tumor_apical	Tumor_lateral	Tumor_basal	Tumor_cytosol	Epi_apical	Epi_lateral	Epi_basal	Epi_cytosol	Stromal	Matrix
Tumor_apical	-	-	5	20	5	-	-	-	-	-	-
Tumor_lateral	-	5	5	5	5	-	-	-	-	-	-
Tumor_basal	-	20	5	5	5	-	-	-	-	-	-
Tumor_cytosol	-	5	5	5	5	-	-	-	-	-	-
Epi_apical	-	-	-	-	-	5	5	20	5	-	-
Epi_lateral	-	-	-	-	-	5	5	5	5	-	-
Epi_basal	-	-	-	-	-	20	5	5	5	-	-
Epi_cytosol	-	-	-	-	-	5	5	5	5	-	-
**Models: Low polarity**											
Contact energy	Medium	Tumor_apical	Tumor_lateral	Tumor_basal	Tumor_cytosol	Epi_apical	Epi_lateral	Epi_basal	Epi_cytosol	Stromal	Matrix
Medium	0	4	30	30	30	4	30	30	30	30	30
Tumor_apical	4	20	10	10	10	20	10	10	10	10	10
Tumor_lateral	30	10	5	10	10	10	5	10	10	10	10
Tumor_basal	30	10	10	10	10	10	10	10	10	10	10
Tumor_cytosol	30	10	10	10	10	10	10	10	10	10	10
Epi_apical	4	80	40	40	40	80	40	40	40	40	40
Epi_lateral	30	40	5	40	40	40	5	40	40	40	40
Epi_basal	30	40	40	40	40	40	40	40	40	3	3
Epi_cytosol	30	40	40	40	40	40	40	40	40	40	40
Stromal	30	40	40	3	40	40	40	3	40	3	1
Matrix	30	40	40	3	40	40	40	3	40	1	1
Internal adhesion energy	Medium	Tumor_apical	Tumor_lateral	Tumor_basal	Tumor_cytosol	Epi_apical	Epi_lateral	Epi_basal	Epi_cytosol	Stromal	Matrix
Tumor_apical	-	5	5	20	5	-	-	-	-	-	-
Tumor_lateral	-	5	5	5	5	-	-	-	-	-	-
Tumor_basal	-	20	5	5	5	-	-	-	-	-	-
Tumor_cytosol	-	5	5	5	5	-	-	-	-	-	-
Epi_apical	-	-	-	-	-	5	5	20	5	-	-
Epi_lateral	-	-	-	-	-	5	5	5	5	-	-
Epi_basal	-	-	-	-	-	20	5	5	5	-	-
Epi_cytosol	-	-	-	-	-	5	5	5	5	-	-
**Models: No polarity**											
Contact energy	Medium	Tumor_apical	Tumor_lateral	Tumor_basal	Tumor_cytosol	Epi_apical	Epi_lateral	Epi_basal	Epi_cytosol	Stromal	Matrix
Medium	0	10	10	10	10	10	10	10	10	10	10
Tumor_apical	10	10	10	10	10	10	10	10	10	10	10
Tumor_lateral	10	10	10	10	10	10	10	10	10	10	10
Tumor_basal	10	10	10	10	10	10	10	10	10	10	10
Tumor_cytosol	10	10	10	10	10	10	10	10	10	10	10
Epi_apical	4	80	40	40	40	80	40	40	40	40	40
Epi_lateral	30	40	5	40	40	40	5	40	40	40	40
Epi_basal	30	40	40	40	40	40	40	40	40	3	3
Epi_cytosol	30	40	40	40	40	40	40	40	40	40	40
Stromal	30	40	40	3	40	40	40	3	40	3	1
Matrix	30	40	40	3	40	40	40	3	40	1	1
Internal adhesion energy	Medium	Tumor_apical	Tumor_lateral	Tumor_basal	Tumor_cytosol	Epi_apical	Epi_lateral	Epi_basal	Epi_cytosol	Stromal	Matrix
Tumor_apical	-	5	5	20	5	-	-	-	-	-	-
Tumor_lateral	-	5	5	5	5	-	-	-	-	-	-
Tumor_basal	-	20	5	5	5	-	-	-	-	-	-
Tumor_cytosol	-	5	5	5	5	-	-	-	-	-	-
Epi_apical	-	-	-	-	-	5	5	20	5	-	-
Epi_lateral	-	-	-	-	-	5	5	5	5	-	-
Epi_basal	-	-	-	-	-	20	5	5	5	-	-
Epi_cytosol	-	-	-	-	-	5	5	5	5	-	-

Table S2 General CC3D-CPM characteristics and simulation parameters in each model, related to Figure 2.

1. CPM: cellular potts model
2. mcs: Monte Carlo steps
3. Contact energy is for the specification of cell-to-cell adhesion energies.
4. Internal adhesion energy is important to establish compartmentalized cells.
5. "NA" means not applicable.
6. The values altered from the base model are highlighted by red color.
7. "-" means not assumed.

Models	Interaction type					Cancer cell-cancer cell connections	Morphological phenotype
	Luminal space and stromal tissue or mucus	Luminal space	Stromal tissue or mucus	No interaction with cancer cells	Interaction only with cancer cells		
Original	+	+++	+++				PPA
3x growth		+++	+++			+	MPA
10x growth		++	+++		+	+++	SPA
1/10 adhesions to stromal tissue		+++	+			+	MPA
1/4 polarity		+++	+++		+	+	SPA
No polarity		++	++		+++	++	SPA
Original + mucus	++	+++	+++				APA
Slow growth + cell death	+	+++	++				PPA
Mild contact inhibition + high cell death rate	+++	+++	+++				LPA

Table S3 Differences of cell-cell interaction modes relate to the morphological phenotypes, related to Figure 2.
LUAD: lung adenocarcinoma, PPA: papillary-predominant adenocarcinoma, MPA: micropapillary-predominant adenocarcinoma, APA: acinar-predominant adenocarcinoma, SPA: solid-predominant adenocarcinoma, LPA: lepidic-predominant adenocarcinoma

LUAD subtype	Function	Highly mutated genes
LPA	Cancer driver	*PIK3CA, TP53, RBM10*
	Cell-cell adhesion	*VANGL2*
	Transcription	*NPAS3, ZNF43, ZNF91, ZNF761*
	Others	*VPS13B*
APA	Cancer driver	*TP53, EGFR*
	Cell-cell adhesion	*ADGRL2*
	Transcription	*CTCF, TAF1L, ZNF43, ZNF100, ZNF569, ZNF696, ZNF697*
	Others	*CGRRF1, CSF1R, CYYR1, FPGT, GIPC3, IGLV3-1, MDC1, NDUFAF6,*
PPA	Cancer driver	*EGFR*
	Cell-cell adhesion	*NA*
	Transcription	*ZNF57, ZNF569, ZNF696, ZNF697, ZNF845*
	Others	*ADAMTS2, ALMS1, ATOH8, FPGT, FPGT-TNNI3K, HLA-B, HLA-DPB1, PPP3CA, ZDHHC11B*
MPA	Cancer driver	*KRAS, STK11, KEAP1*
	Cell-cell adhesion	*ADGRL3, TMTC1*
	Transcription	*ZNF92*
	Others	*ACKR1, ADPRHL1, PCLO, TGOLN2*
SPA	Cancer driver	*TP53, STK11, SMARCA4*
	Cell-cell adhesion	*PCDHGA9*
	Transcription	*NPAS3, ZNF678, ZNF681*
	Others	*ACSS3, ALMS1, CAC02D1, CYYR1, F5, GIMAP6, INPP4B, LILRA6, MAGEE1,*<br>*MUC16, PPP1R12A, PPP3CA, QRICH2, RYR2, TLR4, ZDHHC11B, VSIG4, VWDE*

Table S4 LUAD subtype specific high-rate mutated genes, related to Figure 5.
LUAD: lung adenocarcinoma, PPA: papillary-predominant adenocarcinoma, MPA: micropapillary-predominant adenocarcinoma,

Comparison	Upregulated in	GO term	Genes	Number of total genes	Prognosis	Number of genes	Ratio (%)
MPA vs PPA	MPA	Collagen-containing extracellular matrix	*COL12A1, COL25A1, MFAP5*	6	Positive	1	20.0
					Neutral	0	0.0
		External side of plasma membrane	*ACKR3, ANTXR1, ITGA11*		Negative	4	80.0
SPA vs PPA	SPA	Collagen-containing extracellular matrix	*ADAMTS4, ADAMTS5, ANGPTL2, CDH2, COL11A1, COL12A1, COL15A1, COL1A1, COL1A2, COL3A1, COL5A1, COL5A2, COL7A1, COMP, CTHRC1, FGA, GPC6, GREM1, LAMA1, LOXL2, LRRC15, MFAP5, MXRA5, PXDN, S100A9, SERPINE2, SFRP2, SULF1, THBS2, VTN*	30	Positive	3	10.0
					Neutral	5	16.7
					Negative	22	73.3
	PPA	Apical part of cell	*ACE2, ADGRF5, CD36, DUOX1, DUOX2, DUOXA2, FOLR1, IL6R, MUC1, MUC17, PTCH1, SLC10A2, SLC26A9, SLC34A2, SLC4A5, SLC6A20*	19	Positive	16	84.2
		Apical plasma membrane	*ACE2, CD36, CLIC5, DUOX1, DUOX2, FOLR1, IL6R, MUC1, MUC17, SLC10A2, SLC26A9, SLC34A2, SLC4A5, SLC6A20*		Neutral	0	0.0
		Anchored component of membrane	*CEACAM8, CPM, FOLR1, GPC5*		Negative	3	15.8

Table S5 Cell-cell interaction-related differential gene expression between subtypes, related to Figures 5 and 6

PPA: papillary-predominant adenocarcinoma, MPA: micropapillary-predominant adenocarcinoma, APA: acinar-predominant adenocarcinoma, SPA: solid-predominant adenocarcinoma, LPA: lepidic-predominant adenocarcinoma

Behavior[1] → Cell type ↓	Polarity and Compartments	Volume Constraint	Adhesions	Focal Point Adhesions[2]	Proliferation (contact inhibition)	Necrosis (death, deletion)
**Normal lung epithelial cell**	Apical, basal, lateral, cytosol	yes	yes (internal and external)	Intracellular only	no	Random
**Cancer lung epithelial cell**	Apical, basal, lateral, cytosol	yes	yes (internal and external)	Intracellular only	yes (inhibited when two or more neighboring epithelial cells)	Random
**Stromal Cell**	none	yes	yes	none	yes	no
**Extracellular matrix**	none	yes	yes	none	yes	no
**Mucus**	none	yes	yes	none	yes	no
**Medium**	none	no	yes	none	no	na

Table S6 Capabilities of the cell types in the CPM simulations, related to STAR Methods.

Notes:

1. In a particular simulation, a cell type may have a capability without using it. For example, all cancer cells have a proliferation capability, but parameters of the individual model may disable that capability by having a proliferation rate of zero.
2. Additional distance constraints to maintain the proper spatial relationship between an epithelial cell's compartments.
